# Factors regulated by interferon gamma and hypoxia-inducible factor 1A contribute to responses that protect mice from *Coccidioides immitis* infection

**DOI:** 10.1186/1471-2180-12-218

**Published:** 2012-09-24

**Authors:** Christopher H Woelk, Jin X Zhang, Lorraine Walls, Suganya Viriyakosol, Akul Singhania, Theo N Kirkland, Joshua Fierer

**Affiliations:** 1 Veterans Affairs San Diego Healthcare System, Mail Code 9111-F, San Diego, California, 92161, USA; 2 Department of Medicine, University of California San Diego, La Jolla, California, 92093, USA; 3 Department of Pathology, University of California San Diego, La Jolla, California, 92093, USA

**Keywords:** Coccidioidomycosis, Gene expression, Innate immunity, Interferon, *HIF1A*

## Abstract

**Background:**

Coccidioidomycosis results from airborne infections caused by either *Coccidioides immitis* or *C. posadasii*. Both are pathogenic fungi that live in desert soil in the New World and can infect normal hosts, but most infections are self-limited. Disseminated infections occur in approximately 5% of cases and may prove fatal. Mouse models of the disease have identified strains that are resistant (*e.g.* DBA/2) or susceptible (*e.g.* C57BL/6) to these pathogens. However, the genetic and immunological basis for this difference has not been fully characterized.

**Results:**

Microarray technology was used to identify genes that were differentially expressed in lung tissue between resistant DBA/2 and sensitive C57BL/6 mice after infection with *C. immitis*. Differentially expressed genes were mapped onto biological pathways, gene ontologies, and protein interaction networks, which revealed that innate immune responses mediated by Type II interferon (*i.e.*, *IFNG*) and the signal transducer and activator of transcription 1 (*STAT1*) contribute to the resistant phenotype. In addition, upregulation of hypoxia inducible factor 1A (*HIF1A*), possibly as part of a larger inflammatory response mediated by tumor necrosis factor alpha (*TNFA*), may also contribute to resistance. Microarray gene expression was confirmed by real-time quantitative PCR for a subset of 12 genes, which revealed that *IFNG HIF1A* and *TNFA*, among others, were significantly differentially expressed between the two strains at day 14 post-infection.

**Conclusion:**

These results confirm the finding that DBA/2 mice express more Type II interferon and interferon stimulated genes than genetically susceptible strains and suggest that differential expression of *HIF1A* may also play a role in protection.

## Background

Coccidioidomycosis is one of the endemic mycoses in the New World caused by one of two closely related dimorphic fungi, *Coccidioides immitis* and *C. posadasii*[1]*.* These fungi grow in the arid alkaline soil of the Lower Sonoran Life Zone and infectious arthroconidia are aerosolized by wind and inhaled. Once inside the lung the fungus converts into the pathognomonic spherule under the influence of increased temperature and pCO_2_. It is estimated that 150,000 people are infected each year of which approximately 60% resolve on their own and do not require medical intervention [2,3]. The others have either symptomatic pneumonias or they develop disseminated disease [4]. The risk factors for dissemination are T cell deficiencies such as AIDS, organ transplantation, and pregnancy, as well as treatment with tumor necrosis factor alpha (TNF-α) inhibitors [5,6]. Furthermore, the risk of disseminated coccidioidomycosis is 5–10 times higher for previously healthy African-Americans and Filipinos than for Caucasians [7,8]. This strongly suggests that there is a genetic basis for susceptibility to disseminated coccidioidomycosis. The immune response of patients who develop disseminated coccidioidomycosis is different from those with self-limited infections. The former make high titers of antibody against fungal antigens and do not have positive skin tests (Th2), and conversely, the latter respond to infection with low titers of antibody and skin test reactivity [9]. The genetic basis for the aberrant immune response in susceptible individuals is not clearly defined.

Several years ago we discovered that inbred strains of mice vary over 4 logs in their susceptibility to infection with *C. immitis* and that resistance is the dominant phenotype [10]. This proved to be a polygenic trait, and a resistance locus was identified on chromosome 6 using recombinant inbred BXD lines [11]. C57BL/6 mice are more sensitive to infection with *C. immitis* than DBA/2 mice such that nearly all C57BL/6 mice die between day 16 and 18 post-infection [10]. We have shown that infected C57BL/6 mice make more IL-10 and IL-4 and less interferon gamma (IFN-γ) in their lungs compared to DBA/2 mice [12]. IL-10 has pleiotropic effects on different cell types that affect the acquired immune response resulting in inhibition of the development of Th1 immune responses [13].

In the current work, microarray analysis was used to identify genes differentially expressed between lung tissue samples from resistant DBA/2 and sensitive C57BL/6 mice following infection with *C. immitis*. Differentially expressed genes were mapped onto biological pathways, gene ontologies and protein networks in order to fully characterize the biological processes that contribute to a protective response against *C. immitis* infection.

## Results

### *C. immitis* infection in DBA/2 resistant versus sensitive C57BL/6 mice

**Figure 1:**
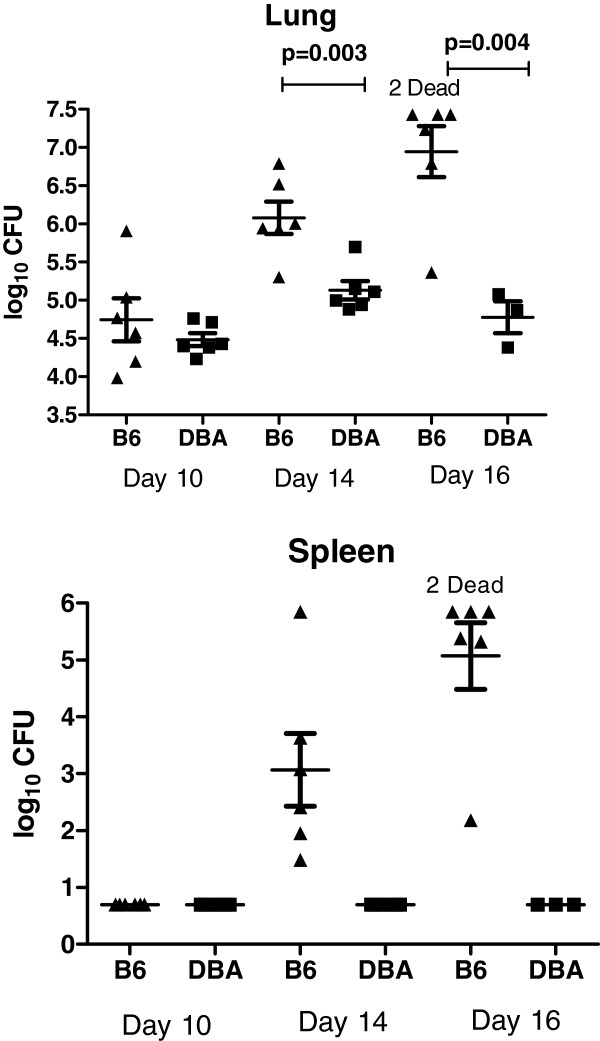
**Comparison of *C. immitis *infection between resistant DBA/2 and sensitive C57BL/6 mice.** Mice were infected (*i.n.*) and then sacrificed at the indicated intervals. The right lung and spleen of each mouse was homogenized and cultured quantitatively. Each symbol represents an individual mouse and the horizontal lines are the geometric mean ± standard error of the mean. The infection proceeded more rapidly in the lungs of the C57BL/6 mice and dissemination to the spleen occurred only in this strain, another indication of their increased susceptibility to infection. *P*-values comparing lung CFU were calculated with an unpaired Student’s *t*-test using GraphPad Prism (San Diego, CA). There was no significant difference between CFU in the lungs of the two strains on day 10 after infection.

The colony forming units (CFUs) in the right (R) lung and spleen of DBA/2 and C57BL/6 mice were determined after intra-nasal (*i.n.*) infection with *C. immitis* arthroconidia. We chose three time points after infection for analysis (day 10, 14 and 16). Since mice were only infected with 50 CFU and not all of them were inhaled, day 10 is the earliest day when there are enough organisms in the lungs to reliably quantitate pulmonary infection in all mice. C57BL/6 mice began to die on day 16 so this was selected as the last time point, and day 14 was chosen as an intermediate time point. On day 10 after infection there were equal numbers of CFU in the lungs of both strains of mice and we could not detect dissemination by culturing their spleens (Figure 1). On day 14 and 16 post-infection DBA/2 mice had 10 to 100 fold fewer CFU/lung, and in this experiment no DBA/2 mice had detectable dissemination to the spleen, whereas all the C57BL/6 mice had positive spleen cultures.

### Microarray analysis of mouse strains with differential resistance to infection with *C. immitis*

**Figure 2:**
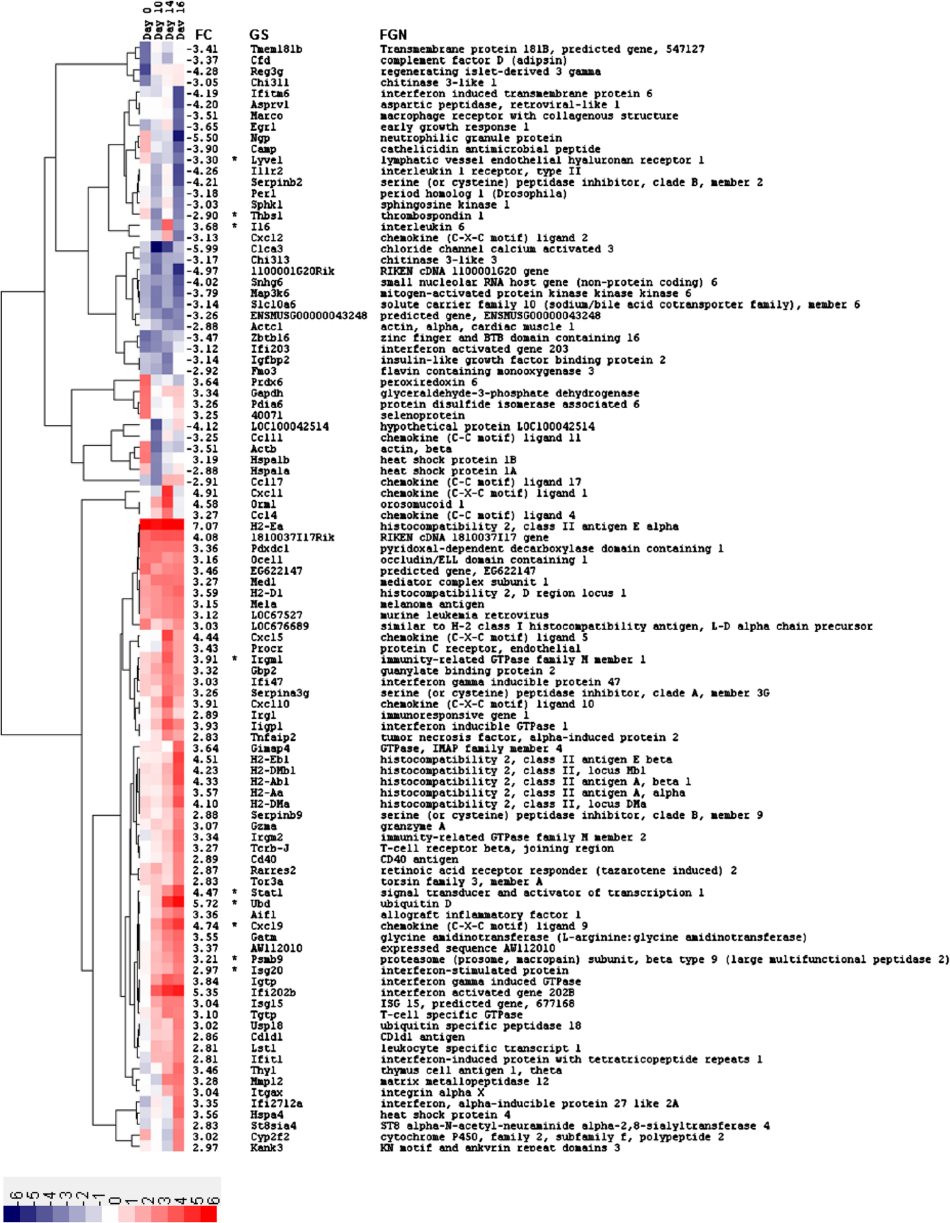
**A heatmap depicting the top 100 modulated genes that were differentially expressed between DBA/2 and C57BL/6 mice.** Fold changes were calculated between mice strains prior to (day 0) and following infection (days 10, 14, and 16) with *C. immitis*. For each gene, the absolute peak log_2_ fold change (FC) was identified across time points and the top 100 included in the heatmap. The log_2_ fold change scale is indicated at the bottom of the heatmap, where red shading indicates greater expression in DBA/2 compared to C57BL/6 mice and blue shading represents lesser expression. For example, red shading will result if a gene is expressed to a greater extent in DBA/2 compared to C57BL/6 mice or if a constitutively expressed gene is downregulated in DBA/2 to a lesser extent compared to C57BL/6. Therefore, the direction of gene expression changes for each of the top 100 modulated genes is presented in Additional file 1: Figure S1 by dividing expression levels at post-infection time points (day 10, 14, and 16) by those in the uninfected control (day 0). Hierarchical clustering of genes based on their expression profiles over the time course is reflected in the dendogram to the right of the heatmap and was performed by calculating distances using the Pearson correlation metric and then clustering distances using the average linkage method. The expression of genes marked with an asterisk (*) was confirmed by RT-qPCR. Annotation columns are as follows: *FC*, peak log_2_ fold change; *GS*, gene symbol; *FGN*, full gene name.

**Figure 3:**
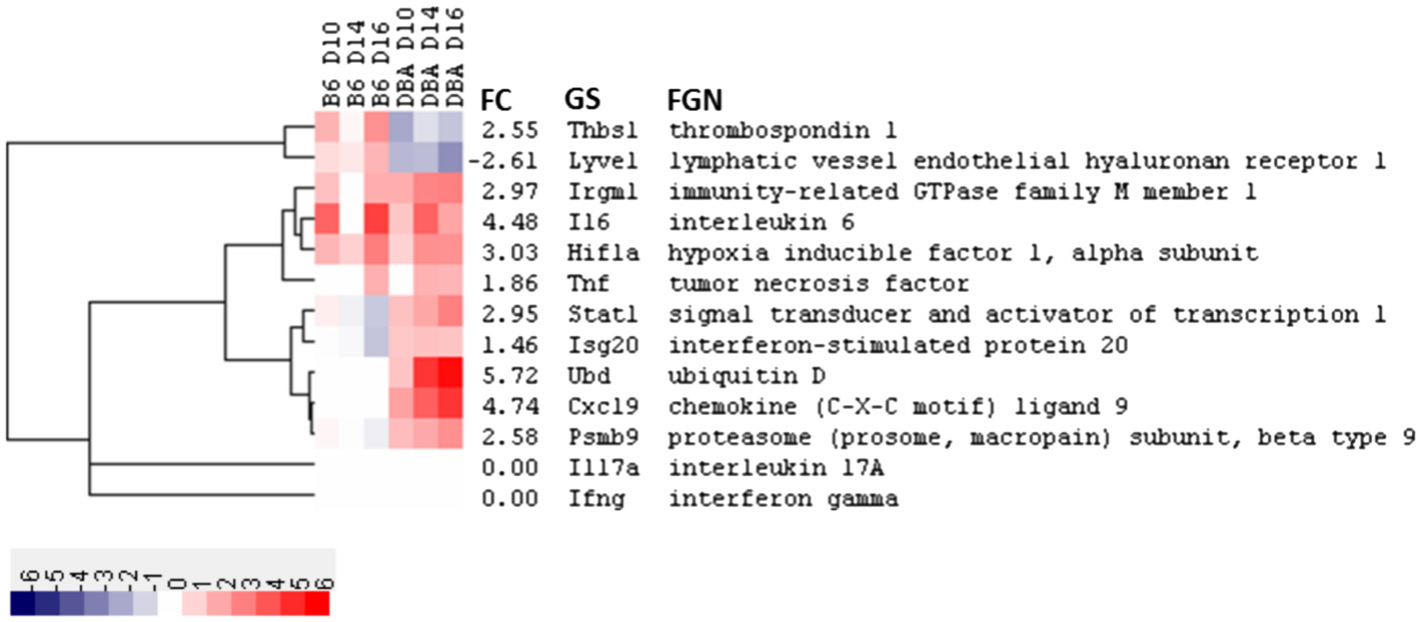
**A heatmap of fold changes calculated by comparing gene expression at post-infection time points to day 0 (pre-infection) for the 13 targets selected for RT-qPCR analysis.** Calculating fold changes in this way provides confirmation of the direction (up or down) of expression changes. Fold change is presented on a log_2_ scale as indicated at the bottom of the heatmap, where red shading indicates upregulation and blue shading represents downregulation of gene expression. The genes were clustered based on their expression profiles as described in the legend for Figure 2. The abbreviations for the annotation columns are defined as for Figure 2.

Genes that were differentially expressed between mouse strains (DBA/2 and C57BL/6) before (day 0) and after (day 10, 14 and 16) infection with *C. immitis* were identified by microarray analysis in an unbiased manner, in order to determine the basis for resistance. A total of 1334 genes were differentially expressed between mice strains with a fold change ≥ 2 or ≤ -2 (log_2_ fold change ≥ 1 or ≤ -1, respectively) for at least one time point. The top 100 of these differentially expressed genes indicated a wide range of different expression profiles over the time course (Figure 2). We focused on those genes that showed no differential gene expression prior to infection (day 0) but were then expressed to different degrees in DBA/2 and C57BL/6 mice after infection. Several genes fitting this profile were related to the innate/acquired immune responses as mediated by IFN [14], and the following IFN-stimulated genes (ISGs) were selected for real-time quantitative PCR (RT-qPCR) analysis: chemokine C-X-C motif ligand 9 (*CXCL9*), immunity-related GTPase family M member 1 (*IRGM1*), interferon stimulated exonuclease gene 20 kDa (*ISG20*), proteosome subunit beta type 9 (*PSMB9*), signal transducer and activator of transcription 1 (*STAT1*) and ubiquitin D (*UBD*). However, the direct interpretation of red for upregulation and blue for downregulation in Figure 2 may be misleading as the color scale reflects the ratio of gene expression in DBA/2 over C57BL/6 mice. Thus a red box in Figure 2 could result either from a gene that was upregulated to a greater extent in DBA/2 than in C57BL/6 mice, or from a gene that was downregulated to a lesser extent (compared to day 0) in DBA/2 compared to C57BL/6 mice (see Materials and Methods). Therefore, fold changes were also calculated by comparing expression levels post-infection (days 10, 14 and 16) to pre-infection levels (day 0) in order to identify the direction of the change in gene expression (Figure 3). This revealed that *CXCL9*, *IRGM1*, *ISG20*, *PSMB9*, *STAT1* and *UBD* at days 10, 14, and 16 were upregulated genes in DBA/2 mice. Post- versus pre-infection fold changes for every gene shown in Figure 2, and not just those selected for RT-qPCR validation (Figure 3), are available in Additional file 1: Figure S1.

Genes expressed to a lesser extent in DBA/2 versus C57BL/6 mice following *C. immitis* infection are also interesting and these too were validated by RT-qPCR (see below). Thrombospondin 1 (*THBS1*) and the lymphatic vessel endothelial hyaluronan receptor 1 (*LYVE1*) fit this profile (Figure 2) and were selected for RT-qPCR analysis. Again, comparison of gene expression between pre- and post-infection time points confirmed these genes were actually more downregulated in DBA/2 mice (Figure 3).

### Pathway and gene ontology analysis

**Figure 4:**
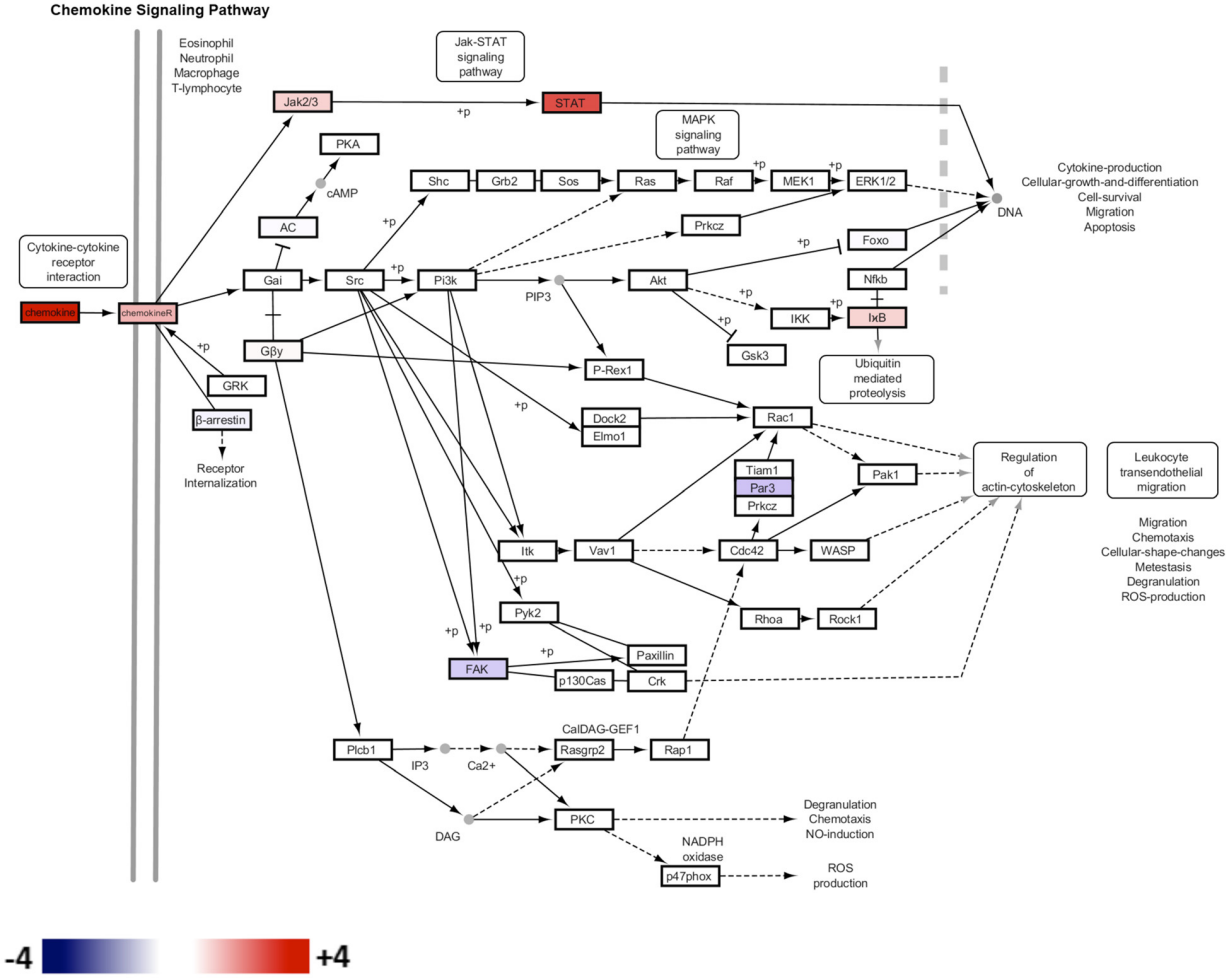
***Chemokine Signaling Pathway *from the KEGG database (ID: mmu04062) overlaid with log_2 _fold change values for genes differentially expressed between DBA/2 and C57BL/6 at day 14.** The scale for log_2_ fold change values is indicated at the bottom of the pathway diagram, where red shading indicates greater expression in DBA/2 compared to C57BL/6 mice and blue shading represents lesser expression. Genes not differentially expressed, *i.e.*, with a fold change between −2 and +2 (log_2_ fold change between −1 and +1) are depicted in white.

We used the Database for Annotation, Visualization, and Integrated Discovery [DAVID [15] to identify pathways that were significantly over-represented in the set of 1334 differentially expressed genes. Four pathways were enriched for differentially expressed genes with a false discovery rate (FDR) corrected *p*-value <0.05, and the majority of these pathways were associated with immune responses (Additional file 2: Table S1). In agreement with the large number of ISGs identified in the top 100 modulated genes (Figure 2), the two pathways containing the greatest enrichment for differentially expressed genes were the *Chemokine signaling* and *Cytokine-cytokine receptor interaction* pathways. The fold changes associated with the differentially expressed genes at day 14 post-infection were superimposed on the *Chemokine signaling* pathway and visualized using Cytoscape (Figure 4). Chemokine signaling clearly contributes to the upregulation of ISGs since the following signaling cascade is upregulated at the transcriptional level: Chemokine → Chemokine receptor (R) → JAK2/3 → STAT → ISG expression (Figure 4).

**Figure 5:**
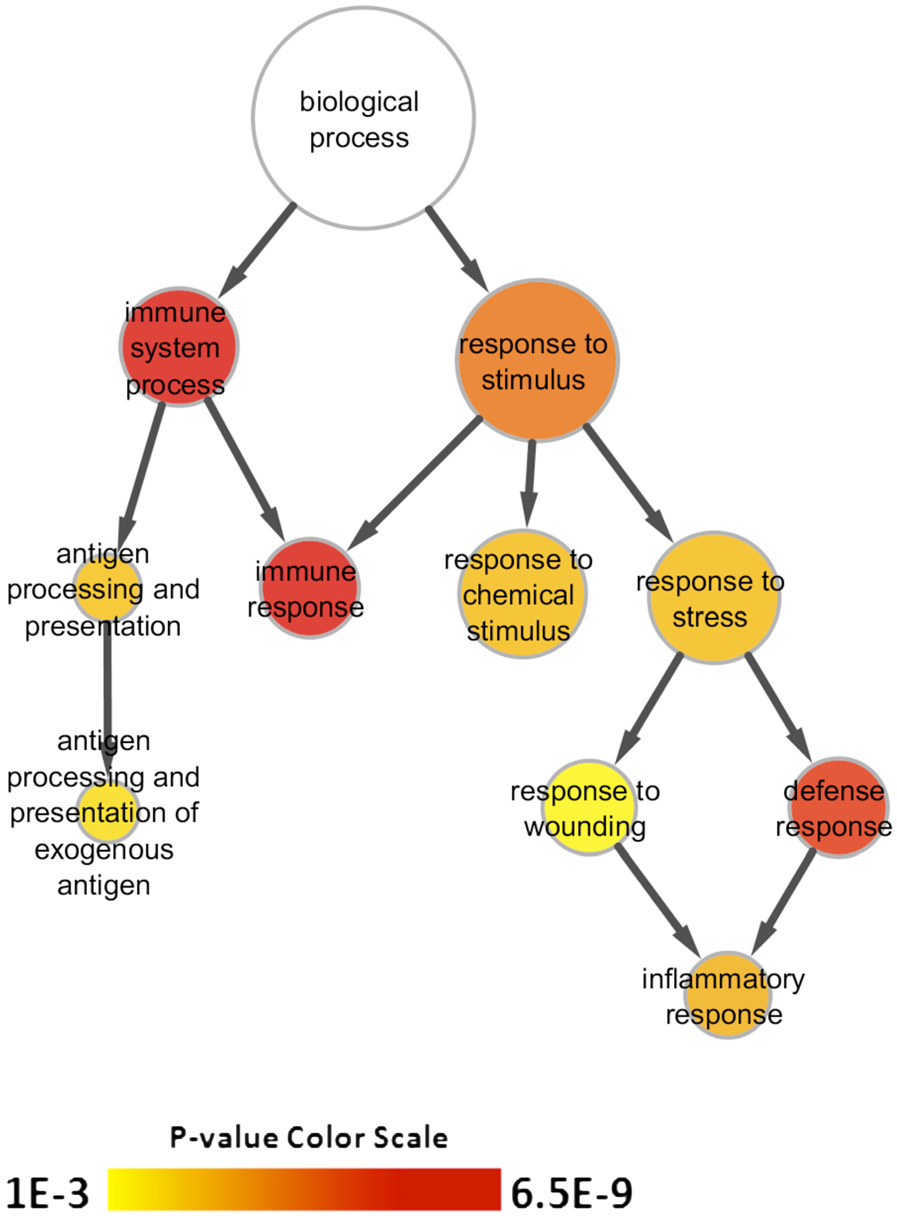
**Hierarchical depiction of GO terms significantly over-represented in the set of genes that were differentially expressed with a fold change ≥ 2 or ≤ -2 (log_2 _fold change ≥ 1 or ≤ -1, respectively) between DBA/2 and C57BL/6 mice at any time point (N = 1334).** The size of the node associated with each GO term is relative to the number of differentially expressed genes belonging to that term. The color scale indicates the level of significance associated with each node with red being the most significant. For display purposes only GO terms with an FDR corrected *p*-value <0.001 are depicted. The full list of significant GO terms using an FDR corrected p-value cut off <0.05 is available in Additional file 2: Table S2.

The identification of gene ontology (GO) terms significantly over-represented in the set of 1334 differentially expressed genes was performed using the Biological Networks Gene Ontology (BiNGO) tool [16], which preserves the hierarchical relationship among ontology terms (Figure 5). Using an FDR corrected *p*-value cut-off <0.001 the three most significant GO terms were: *immune system process*, *immune response*, and *defense response*. Therefore, the immune related terms revealed by GO analysis agree with the results obtained from pathway analysis. The entire list of GO terms that were significantly enriched for differentially expressed genes at an FDR corrected *p*-value <0.05 are available in Additional file 2: Table S2.

### Protein network analysis

**Figure 6:**
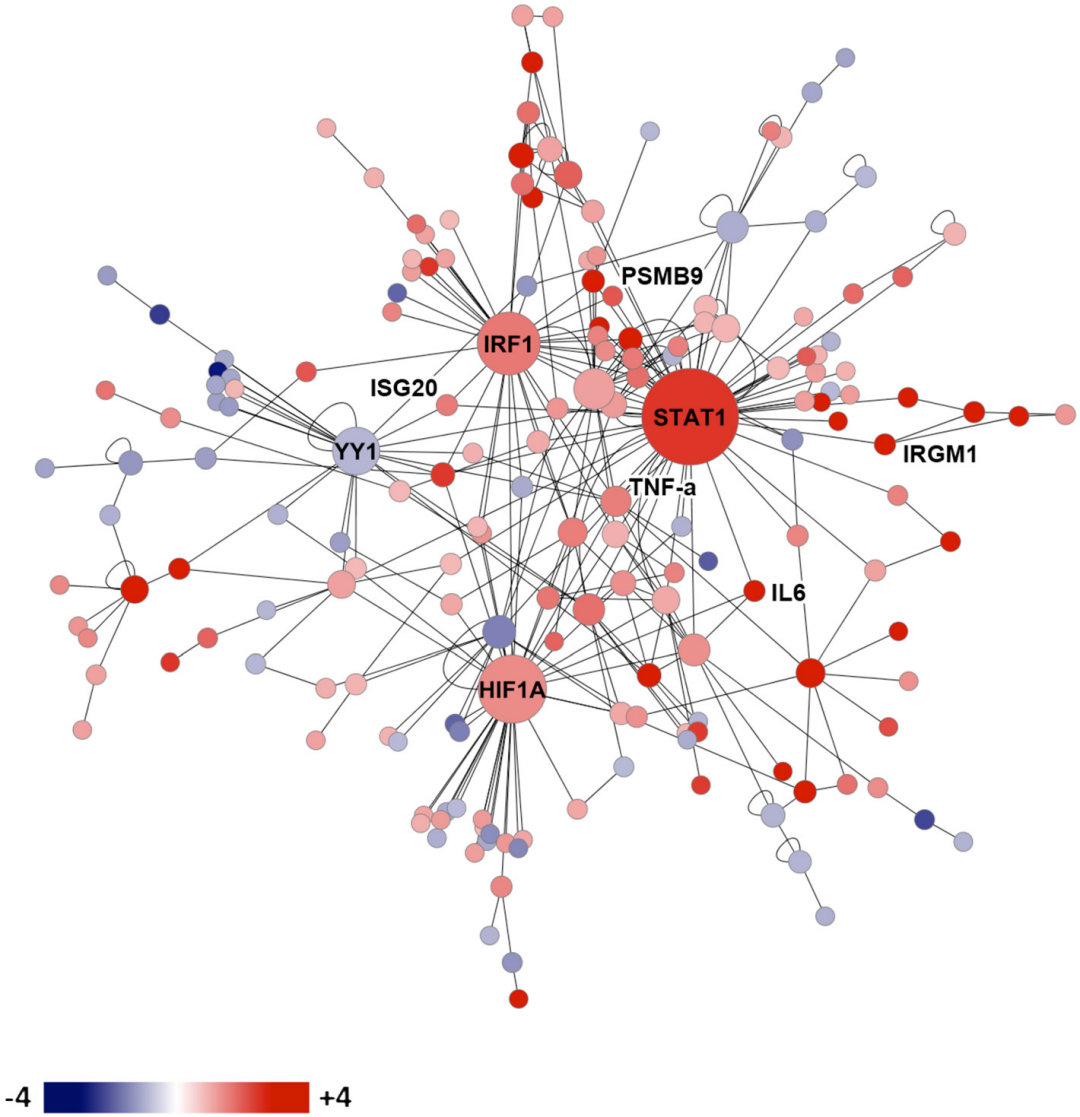
**Direct protein interaction network constructed from the genes differentially expressed with a fold change ≥ 2 or ≤ -2 (log_2 _fold change ≥ 1 or ≤ -1, respectively) between DBA/2 and C57BL/6 mice at day 14 following *C. immitis *infection (N = 416).** MetaCore was used to identify protein-protein and protein-DNA interactions between the protein products of differentially expressed genes and Cytoscape was used to visualize the network. Log_2_ fold changes were superimposed on this protein network such that red indicates greater expression in DBA/2 versus C57BL/6 mice, and blue lesser expression, as indicated by the scale bar. Each node represents a gene and the size of a node is indicative of the number of interactions the product of each gene makes at the protein level. The largest nodes are labeled *HIF1A*, *IRF1*, *STAT1* and *YY1*, and represent hubs that correspond to transcription factors.

Protein-protein and protein-DNA interactions between 416 genes that were differentially expressed between mice strains at day 14 were identified using MetaCore (GeneGo, St. Joseph, MI). The resulting protein interaction network depicted in Figure 6 consists of four major hubs: hypoxia inducible factor 1A (*HIF1A*), interferon regulatory factor 1 (*IRF1*)*, STAT1*, and Yin Yang 1 (*YY1*). These hubs represent transcription factors, which themselves, as well as their targets, were differentially expressed between mouse strains. *HIF1A*, *IRF1*, and *STAT1*, were expressed to a greater extent in DBA/2 compared to C57BL/6 mice, and *YY1* to a lesser extent. *STAT1* is the largest hub representing the transcription factor regulating the most differentially expressed genes and it was previously selected as a target for RT-qPCR confirmation from the top 100 modulated genes (Figure 2). *YY1* is a transcription factor whose “yin-yang” designation reflects its ability to both activate and repress transcription through interactions with histone acetylases and deacetylases, respectively [17]. A novel finding from the protein network analysis was the hub HIF-1α, which is a transcription factor that plays a central role in the cellular and systemic responses to hypoxia. HIF-1α is regulated at the post-translational level, which results in increases in protein half-life, and also at the transcriptional level by NF-κB [18,19]. *HIF1A* was selected for gene expression confirmation by RT-qPCR, as was interleukin 6 (*IL6*), since it is a transcriptional target of both HIF-1α and Stat1 [20,21].

*Stat1* and *Irf1* are both transcription factors that upregulate the expression of ISGs and thus corroborate the presence of ISGs in the top 100 modulated genes (Figure 2), as well as the identification of chemokine related pathways (Figure 4). The well-characterized ISGs selected for RT-qPCR analysis, *IRGM1*, *ISG20* and *PSMB9*[22,23], were targets of Stat1 regulation in protein network analysis (Figure 6). In contrast, *Ubd* (also known as *Fat10*) and *Cxcl9*, were not identified as regulatory targets of STAT1 in protein network analysis. However, they were both retained for RT-qPCR analysis since these genes are clearly regulated by IFN-γ as previously demonstrated using promoter/reporter gene constructs in the case of *Ubd*[24,25] and gene expression studies in the case of *Cxcl9*[26].

### Targeted cytokine array analysis

Microarray gene expression (Figure 2), pathway (Figure 4), and protein network (Figure 6) analysis, clearly indicated that interferon-mediated innate immune responses are upregulated in DBA/2 mice and thus are associated with resistance to *C. immitis* infection. The upregulation of the ISGs *CXCL9* and *UBD* in DBA/2 mice, which are predominantly modulated by Type II IFN [14,27,28], suggested that the interferon gamma (*IFNG*) gene should also be upregulated in this mouse strain. However, *IFNG* was not a top 100 modulated gene (Figure 2) and upon closer examination of the microarray data was found to be expressed below background levels (data not shown). Since our initial time course may have missed the peak of induction of *IFNG*, a targeted analysis of cytokine expression was performed at an additional time point (day 15) using a complementary technology, namely the Mouse Common Cytokines Gene Array from SABiosciences (Frederick, MD, USA). This cytokine array confirmed that *IFNG* was expressed to a greater extent in DBA/2 compared to C57BL/6 mice with a log_2_ fold change of 1.50 (actual fold change of 2.82, Additional file 1: Figure S2). The cytokine with the greatest differentially expression between mice strains at day 15 detected by the Mouse Common Cytokines Gene Array was interleukin 17A (*IL17A*), which had a log_2_ fold change of 1.83 (actual fold change of 3.56). Therefore, *IFNG* and *IL17A* were also selected as targets for RT-qPCR analysis at days 14 and 16 in order to determine if this more sensitive technique could confirm expression of these cytokines at these time points.

### Real-time quantitative PCR analysis of interferon and hypoxia associated genes

**Figure 7:**
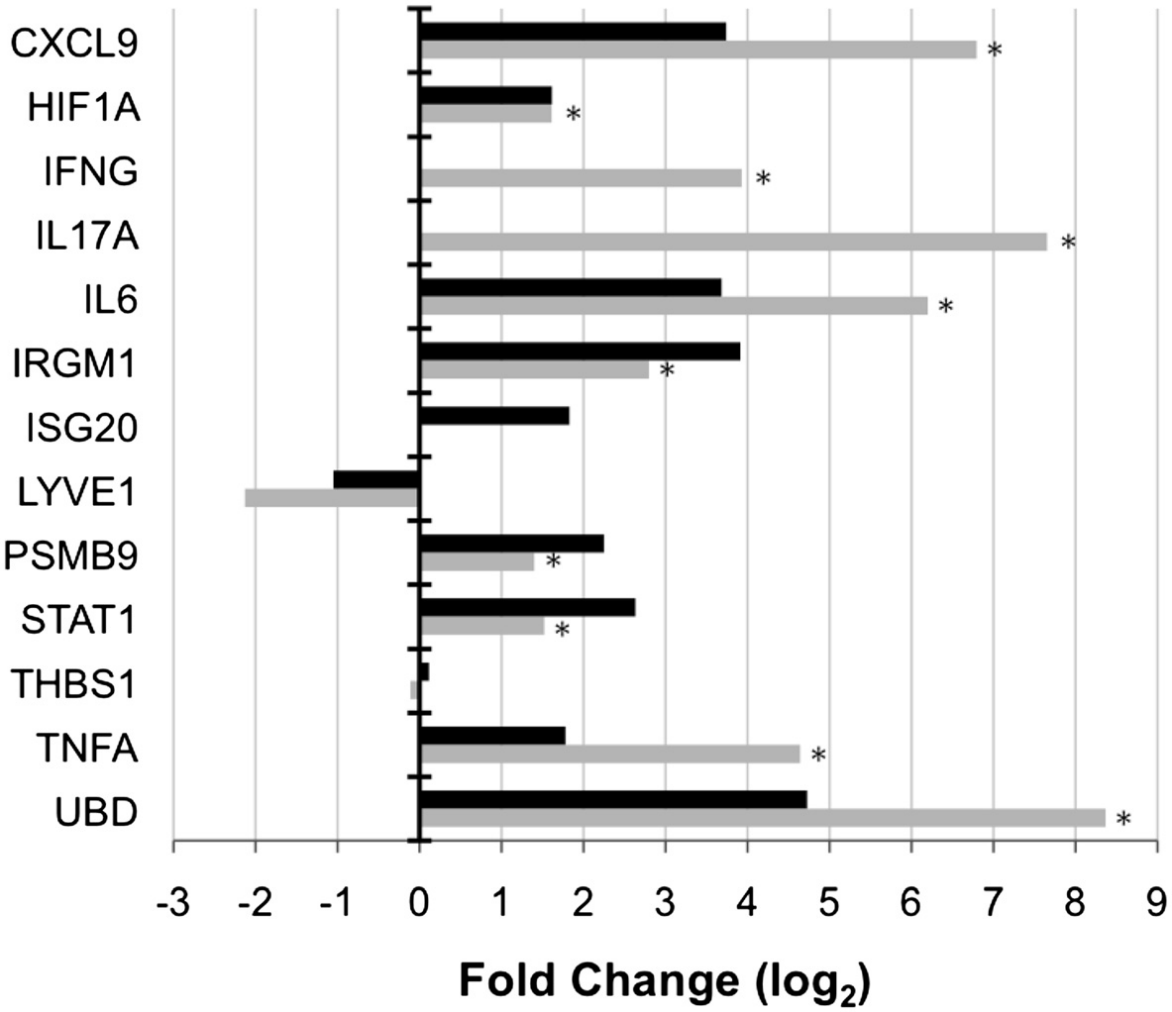
**Confirmation of gene expression differences by RT-qPCR between DBA/2 and C57BL/6 mice at day 14 following *C. immitis *infection.** The fold change for each gene, calculated by dividing the expression level in DBA/2 mice by the expression level in C57BL/6 mice is presented for RT-qPCR data (grey bars). These fold change differences are also compared to microarray results (black bars) for each gene. For the RT-qPCR data, gene expression was assessed using 2 independent samples from C57BL/6 mice and 3 independent samples from DBA/2 mice. RT-qPCR gene expression data (2^-∆∆CT^) was averaged within mouse strains and then used to calculate log_2_ fold change values between strains for direct comparison to microarray data. A log_2_ fold change of 1 equates to an actual fold change of 2. A positive fold change indicates the gene was expressed to a greater extent in DBA/2 mice, and a negative fold change means higher expression in C57BL/6. An asterisk (*) indicates that the gene was significantly differentially expressed (*p* <0.05, *t*-test) between mice strains at day 14.

To validate microarray gene expression results and further confirm the role of responses to IFN-γ and HIF-1α in the resistance of DBA/2 mice to *C. immitis* infection, RT-qPCR analysis was performed at days 10 (Additional file 1: Figure S3A), 14 (Figure 7), and 16 (Additional file 1: Figure S3B) post-infection for the following thirteen targets: *CXCL9*, *HIF1A*, *IFNG, IL6*, *IL17A*, *IRGM1*, *ISG20*, *LYVE1*, *PSMB9*, *STAT1, THBS1*, *TNFA* and *UBD*. The differential gene expression between mice strains detected by microarray was confirmed at day 14 by RT-qPCR for all targets at the 2-fold level (log_2_ fold change of 1) except for *ISG20*. In addition, although microarray analysis did not indicate that *IFNG* and *IL17A* were differentially expressed between mice strains, RT-qPCR analysis confirmed that both were expressed to a greater extent in DBA/2 compared to C57BL/6 mice at day 14 post-infection with *C. immitis*. Even with a limited number of biological replicates at day 14, the majority of targets (*CXCL9*, *HIF1A*, *IFNG*, *IL17A*, *IL6*, *IRGM1*, *PSMB9*, *STAT1*, *TNFA* and *UBD*) were significantly differentially expressed (*p* <0.05, *t*-test) between mouse strains (Figure 7).

## Discussion

Analysis of the gene expression differences between mice strains resistant (DBA/2) and sensitive (C57BL/6) to infection with *C. immitis* identified a large number of ISGs associated with putative control of this fungal pathogen. Innate/adaptive immune responses as mediated by Type II interferon (IFN-γ) have previously been associated with resistance to infection with *C. immitis*[29,30]. For example, Magee and Cox [29] found that IFN-γ protein levels as measured by ELISA were significantly elevated in DBA/2 mice compared to another susceptible strain (BALB/c) following infection with *C. immitis*. Furthermore, treatment of DBA/2 mice with an anti-IFN-γ monoclonal antibody resulted in a significant decrease in their ability to control this fungal pathogen after pulmonary challenge. This current study expands on their work by clearly demonstrating that downstream ISGs are expressed to a greater extent in resistant DBA/2 compared to sensitive C57BL/6 mice (Figures 2 and 7) and that these genes are modulated by the JAK/STAT pathway (Figures 4 and 6), most likely activated by IFN-γ (Figure 7). These findings are highly relevant to human infection since patients with congenital deficiencies of IFN-γ and the interleukin 12 receptor beta 1 (IL-12rβ1) are susceptible to disseminated coccidioidomycosis [30,31].

The upregulation of ISGs (*i.e. CXCL9, IRGM1*, *PSMB9*, *STAT1* and *UBD*) in DBA/2 compared to C57BL/6 mice was confirmed by RT-qPCR at all days post-infection (Figure 7 and Additional file 1: Figure S3). *STAT1* is integral to JAK/STAT signaling triggered by Type I and II IFN and upregulates a number of ISGs that are involved with the host defense against pathogen infection [32]. *UBD* was the ISG that exhibited the greatest upregulation in DBA/2 mice (Figures 2 and 7), and is induced to a greater extent by IFN-γ than IFN-α in human immune and non-immune cells [14]. Several roles have been ascribed to UBD including targeting proteins for proteosomal degradation [33], activation of the nuclear factor of kappa light polypeptide gene enhancer in B-cells 1 (NF-κB) [34], which is a central mediator of innate immunity, as well as a functional involvement in the programmed cell death mediated by TNF-α in the murine B8 fibroblast cell line [35]. *CXCL9* (or *MIG*) is predominantly upregulated by IFN-γ but may also be activated by Type I IFNs [27] and binds the CXCR3 receptor, which is expressed on T cells in order to promote Th1 responses [36]. *PSMB9*, encoded in the major histocompatibility complex class II region, is another gene inducible by both Type I and II IFNs and is a constituent of the immunoproteosome [37-39]. This gene facilitates a link between the innate and adaptive immune response since site directed mutagenesis studies have revealed a role for PSMB9 in antigen processing and presentation [40]. PSMB9 was the only ISG that was expressed at significantly higher levels in DBA/2 mice at both day 10 (Additional file 1: Figure S3A) and 14 (Figure 7), which suggests that the protein product of this gene may play a key role in resistance to *C. immitis* infection.

*IRGM1* is particularly noteworthy since it belongs to a family of immunity-related GTPases whose other members, *IRGM2* and *IRGM3* (or *IGTP*), were also expressed to a greater extent in resistant DBA/2 compared to susceptible C57BL/6 mice (Figure 2). *IRGM1*-deficient mice are more susceptible to infection with *Mycobacterium tuberculosis*, *M. avium*, *Listeria monocytogenes* and *Salmonella enterica* serovar Typhimurium, as assessed by both mouse survival and bacterial loads in tissues, whereas *IRGM3*-deficient mice exhibit normal resistance [41,42]. In contrast, both *IRGM1* and *3* are required for IFN-γ modulated control of *Toxoplasma gondii* in murine macrophages [43]. It appears that *IRGM1* is critical for normal motility of activated macrophages in mouse models suggesting a pivotal role for this protein in the innate response to infection *in vivo*[44]. The relevance of the IRGM family to human coccidioidomycosis is unclear because the single gene in this family in humans, *IRGM*, is considerably truncated and is not regulated by IFN-γ [41]. However, IRGM does play a role in human innate immunity since it is necessary for the execution of the autophagic pathway in macrophages and the control of intracellular *Mycobacteria*[45].

Greater expression of *IFNG* and *IL17A* were detected in DBA/2 mice at day 15 post-infection using the Mouse Common Cytokines Gene Array (Additional file 1: Figure S2). It was therefore surprising that microarray analysis did not detect differential expression of these cytokines between mice strains at days 14 and 16 (Figures 2 and 3), but RT-qPCR analysis was able to do so (Figure 7 and Additional file 1: Figure S3). It is unclear why microarray analysis was unable to detect the expression of these cytokines especially since *IFNG* expression had been detected using the same array platform (MGU74Av2) in lung tissue from C57BL/6 mice exposed to lipopolysaccharide (LPS) [46]. This array platform was designed using the C57BL/6 genome and thus it is possible that these cytokines were not detected because they were not expressed to high levels in C57BL/6 by *C. immitis* infection and contained too many polymorphisms in the DBA/2 homologues for efficient hybridization. What is clear from the RT-qPCR result is that *IFNG* and *IL17A* are expressed to a greater extent in DBA/2 compared to C57BL/6 mice. The upregulation of *ISG20* in DBA/2 mice originally identified by microarray analysis was also not confirmed by RT-qPCR analysis (Figure 7). The probe set on the microarray (103432_at) and the TaqMan assay (Mm00469585_m1) for *ISG20* (NM_001113527) target different regions of this transcript (*i.e.* 2^nd^ and 3^rd^ versus 1^st^ and 2^nd^ exons, respectively) so alternative splicing could account for the discrepancy [47].

*C. immitis* infection also resulted in the downregulation of genes in DBA/2 versus C57BL/6 mice (Figures 2 and 3), which was confirmed by RT-qPCR (Figure 7, S3A and S3B). *THBS1* encodes thrombospondin, an extracellular protein that binds a large number of substrates (calcium, heparan sulfate, integrins, the CD36 macrophage scavenger receptor, and transforming growth factor beta 1 [TGF-β]) to modulate cellular attachment, migration, differentiation, and proliferation [48]. IFN-γ appears to regulate *THBS1* at the post-transcriptional level in keratinocytes and downregulates *THBS1* mRNA in conjunction with TNF-α [28]. *THBS1*-deficient mice have spontaneous pneumonia that leads to pulmonary hemorrhage, macrophage infiltrations and permanent damage to the lungs, which suggests that this protein is important for maintaining normal pulmonary homeostasis by limiting the extent and/or duration of inflammation [48]. Therefore, it is possible that the downregulation of *THBS1* at day 16 in DBA/2 mice facilitates inflammatory responses that contribute to resistance to *C. immitis* infection, but may also contribute to the long term damage to the lung of DBA/2 mice that eventually leads to their death [49]. Downregulation of *LYVE1* in DBA/2 versus C57BL/6 mice is also consistent with a stronger inflammatory response in DBA/2 mice following *C. immitis* infection. Johnson *et al.*[50] previously demonstrated that an inflammatory response induced in primary human dermal lymphatic endothelial cells through treatment with TNF-α led to the downregulation of *LYVE1* at the transcriptional level. The *LYVE1* gene codes for a type I integral membrane receptor that was thought to function in hyaluronan clearance and hyaluronan-mediated leukocyte adhesion, although this biological role has not been confirmed in knockout mice [50,51]. Consistent with the role of TNF-α in modulating expression of both of these genes (*THBS1* and *LYVE1*) we found that TNF-α was more highly expressed in DBA/2 mice at day 14 by both microarray (fold change of 3.43, data not shown) and RT-qPCR analysis (Figure 7).

Protein interaction network analysis identified the transcription factor *HIF1A* as a network hub. *HIF1A* was upregulated to a greater extent at day 14 in resistant DBA/2 versus susceptible C57BL/6 mice, and this was confirmed by RT-qPCR (Figure 7). Previously it was thought that the only regulation of HIF activity was the result of hypoxia increasing HIF-1α levels post-transcriptionally by inhibition of proteosomal degradation of the protein following prolyl hydroxylation [52], but NF-κB also induces *HIF1A* expression at the transcriptional level [53]. Since TNF-α can stimulate NF-κB activity [54], this implies there is cross talk between NF-κB, TNF-α, and HIF-1α, even under normoxic conditions. Since both mouse strains had pneumonia and we did not measure oxygen saturations, we cannot exclude an influence of a hypoxia-induced increase in HIF-1α in the lungs of both strains after infection. However, C57BL/6 mice were clearly afflicted with more extensive lung disease (Figure 1) so this strain might be expected to mount a stronger hypoxic response leading to higher levels of HIF-1α. Since there was more expression of *HIF1A* mRNA in DBA/2 mice at day 14, it appears that the stronger induction of *HIF1A* in DBA/2 mice may be independent of hypoxia. Hypoxia and inflammation occur in human patients infected with *C. immitis*[55,56] and both those conditions are known to increase levels of the HIF-1α protein [19]. It is quite likely that hypoxia and inflammation act synergistically to increase the level of HIF-1α in this infection, as it has in other models of infection in mice [57].

Cox and Magee [58] noted that spleen cells from DBA/2 mice previously infected with *C. immitis* and stimulated with formalin-killed spherules produced higher levels of TNF-α than C57BL/6 mice. Furthermore, our previous studies have shown that TNF-α deficient mice cannot be successfully immunized with a live, attenuated vaccine strain of *C. immitis*[59]. Given the central role of TNF-α in the inflammatory response it is not surprising that the inhibition of this cytokine is a risk factor for the dissemination of *C. immitis* in human patients [6]. These observations suggest that TNF-α plays a beneficial role in resistance to coccidioidomycosis, perhaps through activation of NF-κB and HIF-1α. Encouragingly, *TNFA*, *HIF1A* and a transcriptional target of *HIF1A* (*IL6*) were all upregulated to a greater extent in DBA/2 compared to C57BL/6 mice at day 14 (Figure 7). This suggests the following activation cascade: *TNFA* → NF-κB → *HIF1A* → *IL6;* where NF-κB is primarily regulated at the protein level by degradation of inhibitory IkB proteins and not upregulated at the transcriptional level [60]. However, this result must be interpreted with care since by day 16, *TNFA*, *HIF1A*, and *IL6* are upregulated in C57BL/6 mice to a greater extent than in DBA/2 mice (Figure 3 and Additional file 1: Figure S3B). Cytokines promoting Th17 development (*i.e.*, TGF-β, IL-6, and IL-1β) and those secreted from Th17 cells (*i.e.*, IL-17a) [61] exhibited a similar pattern of gene expression, *i.e.*, upregulated in DBA/2 at day 14 followed by a receding difference (*TGFB*, *IL1B*, and *IL17A*) or a reversal in differential expression (*IL6*) at day 16 (Figure 7, Additional file 1: Figure S3, and data not shown). Recently Cole et al. [62] demonstrated that loss of a functional Th17 receptor resulted in decreased protection of C57BL/6 mice immunized with an attenuated mutant vaccine of *C. posadasii* and subsequently challenged with a virulent strain. It is plausible that an early inflammatory response coupled with the development of Th17 immune responses at day 14 contributes to the resistance of DBA/2 to infection with *C. immitis*. However, it is plausible that by day 16 there was so much infection in C57BL/6 lungs that IL-6 and TNF-α levels increased so that they were more highly expressed in C57BL/6.

## Conclusions

In summary, the immune response as mediated by Type II IFN (*i.e.*, IFN-γ) is clearly greater in the strain of mice that better controlled *C. immitis* infection. This adds support to the anecdotal report of successful treatment of patients suffering from coccidioidomycosis with IFN-γ therapy [63]. Modulation of HIF-1α responses that are associated with inflammation and hypoxia may also contribute to the resistance of DBA/2 mice to this fungal pathogen. Future work will focus on a more finely graded time course in order to fully characterize the genes differentially expressed between DBA/2 and C57BL/6 mice strains. Recently, deep sequencing methods (*e.g.* SAGE-Seq and RNA-Seq) have been proposed to analyze the expression of genes in the entire transcriptome [64]. While RNA-Seq analysis would not change the central findings of this paper, it is a more sensitive digital technique that might identify a greater number of genes, as well as alternatively spliced variants, that may be differentially expressed between DBA/2 and C57BL/6 mice.

## Methods

### Mice and fungal strains

C57BL/6 and DBA/2 mice were purchased from the Jackson Laboratory (Bar Harbor, ME). Arthroconidia from *C. immitis* (RS strain) were harvested as previously described [65], suspended in buffered saline and kept at 4°C prior to infecting the mice. All animal experiments were approved by the Institutional Animal Care and Use Committee at the VA Medical Center, San Diego.

### Infection of mice with *C. immitis*

Twenty-four mice from each strain (C57BL/6 and DBA/2) were infected *i.n.* with 50 arthroconidia of *C. immitis*. One additional mouse per strain was used as an uninfected control. Eight mice from each strain were sacrificed at either day 10, 14, or 16 post-infection. Lungs and spleens were rapidly removed and one lobe of the left lung was immediately minced and frozen in liquid nitrogen and stored at −70°C. The right lung and spleen were homogenized in 1 mL of sterile saline and serially diluted in saline for quantitation of CFUs using Sabouraud agar.

### RNA isolation and hybridization to microarray

RNA was extracted from frozen lung tissue using the ULTRASPEC^TM^ Total RNA Isolation Kit (Biotecx Labs, Houston, TX). RNA quality was confirmed using agarose gels and concentration determined using a spectrophotometer. For each strain, three mice were chosen that had numbers of CFU closest to the median calculated for the eight mice in that strain at a particular time point, and equal amounts of RNA from the lung of each of the three mice were pooled for microarray gene expression analysis. Double-stranded cDNA was synthesized from RNA isolated using the MessageAmp^TM^ aRNA Kit (Ambion, Austin, TX). Biotinylated cRNA was *in vitro* transcribed from double-stranded cDNA template using MegaScript High-Yield Transcription Kit (Ambion). Resulting cRNA (15 μg) was purified using the MessageAmp^TM^ aRNA Kit and fragmented before hybridization to Affymetrix GeneChip MGU74Av2 microarrays (12,488 probes). GeneChips were washed and stained with streptavidin phycoerythrin according to manufacturer’s instructions prior to scanning with an Agilent Gene Array scanner.

### Microarray data analysis

Quality control analysis of microarray gene expression data was performed as recommended by Bolstad et al. [66]. Briefly, microarray data quality was assessed using the following plots: box, histogram, MA, RNA degradation, housekeeping gene, Relative Log Expression (RLE) and Normalized Unscaled Standard Error (NUSE). None of the microarrays were found to be significant outliers and unsupervised clustering of microarrays revealed no significant batch effects. In addition, physical chip images revealed no manufacturing or spatial artifacts. In short, all microarrays passed quality control checks and were retained for further analysis. Microarray gene expression data was deposited at the Gene Expression Omnibus (http://www.ncbi.nlm.nih.gov/geo/) at the National Center for Biotechnology Information with accession number GSE40379.

Microarray data was transformed to the log_2_ scale and normalized using the GC Robust Multichip Average (GCRMA) method [67]. Fold changes were initially calculated by dividing expression levels in DBA/2 mice by those in C57BL/6 mice at each time point (day 0, 10, 14, and 16). A positive ratio indicates greater expression in DBA/2 mice compared to C57BL/6 mice but does not necessarily equate to upregulation. For example, a gene might be constitutively expressed prior to infection (day 0) in both strains and then following infection downregulated less in DBA/2 mice compared to C57BL/6 mice. This would result in a positive ratio indicative of higher expression in DBA/2 than C57BL/6 even though the gene is downregulated compared to the uninfected control (day 0). Therefore, fold changes were also calculated by dividing post-infection time points (day 10, 14 and 16) by the uninfected control (day 0) in order to confirm the direction of gene expression changes. In addition, abnormally high fold change values may result when expression levels below the limit of detection are used as the denominator in fold change calculations. The limit of detection for this study was calculated as an expression level of 35.3, which was the 95^th^ percentile expression level of the absent and marginal probes identified using the MAS 5 algorithm from Affymetrix [68]. Spurious fold change values were avoided by setting every probe set with an expression level below 35.3 to 35.3.

Cytokine gene expression was further assayed using the GEArray^TM^ Q series Mouse Common Cytokines Gene Array from SABiosciences (Frederick, MD). Three DBA/2 and three C57BL/6 mice were infected *i.n.* with *C. immitis* RS strain and the lungs harvested, as described above, 15 days after infection. RNA was extracted from each mouse as previously described and pooled within strains. RNA was used to generate cDNA probes that were then hybridized to GEArray^TM^ Q series platform and detected by chemiluminescence. Gene expression levels were normalized to the housekeeping gene *GAPDH*. The limit of detection of this platform was taken as twice the expression level of the blank negative control [69], and any gene whose expression was below this limit was subsequently set to this limit in order to avoid spurious fold change calculations. Fold changes were again calculated by dividing gene expression levels in DBA/2 mice by expression levels in C57BL/6 mice for each cytokine.

### Pathway, gene ontology, and protein network analysis

Genes were selected for GO and pathway analysis if they were modulated greater than two-fold (log_2_ fold change ≥ 1 or ≤ -1) between DBA/2 and C57BL/6 mice at any time point. Pathway analysis was performed using DAVID [15] with the background defined as all of the probes on the Affymetrix MGU74Av2 GeneChip. A hypergeometric test was used to identify those pathways from the Kyoto Encyclopedia of Genes and Genomes (KEGG) database that were considered significantly over-represented in the list of differentially expressed genes [70]. Only those pathways with an FDR corrected *p*-value of <0.05 using the Benjamini and Hochberg (BH) method were considered significant [71].

GO analysis was performed using the BiNGO tool [16], which is available as a plug in to Cytoscape [72]. BiNGO was used to retrieve the GO annotation and preserved the hierarchical relationship of GO terms for genes differentially expressed between mouse strains. A hypergeometric test was used to identify those GO terms that were significantly over-represented in the set of differentially expressed genes compared to a background of the entire Affymetrix MGU74Av2 GeneChip. Similar to pathway analysis, the FDR associated with multiple testing was corrected using the BH method [71].

Protein-protein and protein-DNA interactions made between the protein products of the genes that were differentially expressed between mouse strains greater than two-fold (log_2_ fold change ≥ 1 or ≤ -1) at day 14 (N = 416) were determined using the direct interactions algorithm in MetaCore (GeneGo, St. Joseph, MI). The interactions documented in MetaCore have been manually curated and are supported by citations in the literature record. When the proteins encoded by genes form well-connected clusters it is quite likely that they share a common functional response. When protein networks are constructed they often reveal hubs, which represent transcription factors that control the regulation of multiple target genes.

### Real-time quantitative PCR

RT-qPCR using TaqMan® Gene Expression Assays (Life Technologies, Carlsbad, CA) was performed for the following 13 targets in order to confirm microarray gene expression results: *CXCL9* (Mm00434946_m1), *HIF1A* (Mm00468878_m1), *IFNG* (Mm01168134_m1), *IL17A* (Mm00439619_m1), *IL6* (Mm01210733_m1), *IRGM1* (Mm00492596_m1), *ISG20* (Mm00469585_m1), *LYVE1* (Mm00475056_m1), *PSMB9* (Mm00479004_m1), *STAT1* (Mm00439531_m1), *THBS1* (Mm01335418_m1), *TNFA* (Mm99999068_m1) and *UBD* (Mm00499179_m1). Total RNA was isolated from frozen lung tissues of individual DBA/2 and C57BL/6 mice at each time point using the ULTRASPEC^TM^ Total RNA Isolation Kit according to the manufacturer’s instructions (Biotecx Labs). cDNA was reversed transcribed from extracted RNA using the qScript cDNA SuperMix from Quanta Biosciences (Gaithersburg, MD). RNA quality was assessed using the Experion bioanalyzer from Bio-Rad (Hercules, CA). Three C57BL/6 samples (one at day 14 and two at day 16) were determined to be of low quality. Therefore, gene expression of the 13 targets was assessed by RT-qPCR in a total of 15 samples: three samples from both strains at day 10, two C57BL/6 and three DBA/2 samples at day 14, and one C57BL/6 and three DBA/2 samples at day 16.

RT-qPCR was performed with the 7900HT Fast Real-Time PCR System (Life Technologies) using 50 ng of cDNA in a 20 μL reaction volume for each target in duplicate. The reaction conditions were as follows: 50°C for 2 minutes, 95°C for 10 minutes, followed by 45 cycles at 95°C for 15 seconds, and 60°C for 1 minute. RT-qPCR data analysis was performed using DataAssist software (Life Technologies) and the significance of differential gene expression between mouse strains assessed with a *t*-test. Changes in gene expression levels were assessed through relative quantification (RQ) using the endogenous control, glucuronidase beta (*GUSB*, Mm01197698_m1), because it is one of the most stable housekeeping genes found expressed the mouse lung [73]. Briefly, the threshold cycle of amplification (Ct) for each sample was compared with that of the endogenous control *GUSB*. The difference in Ct between the sample and *GUSB* was expressed as ΔCt. For each gene assayed, the difference in ΔCt between each sample and the sample selected as the control (a randomly selected C57BL/6 mouse sample analyzed at each day) was expressed as ΔΔCt. The RQ of each sample was then calculated as 2^-∆∆CT^. RQ values were log_2_ transformed and averaged across biological replicates separately for each time point (day 10, 14 or 16) in order to calculate fold change differences between DBA/2 and C57BL/6 mice for comparison to microarray data. This transformation was also performed prior to statistical analyses with DataAssist in order to satisfy the normality assumption, as previously described [74,75].

## Competing interests

The authors declare that they have no competing interests.

## Authors’ contributions

JF, CHW designed the experiments, supervised the research and wrote the paper, TNK contributed reagents and wrote the paper, LW, SV, JXZ, and AS did experiments and/or data analysis.

## Supplementary Material

Additional file 1 **Figure S1.** A heatmap depicting log_2_ fold changes between pre- (Day 0) and post- (Days 10, 14 and 16) infection time points for the top 100 modulated genes depicted in Figure 2. The log_2_ fold change scale is indicated at the bottom of the heatmap, where red shading indicates upregulation post- versus pre-infection and blue shading represents downregulation. Hierarchical clustering of genes based on their expression profiles over the time course was performed by calculating distances using the Pearson correlation metric and then clustering these distances using the average linkage method. The expression of genes marked with an asterisk (*) was confirmed by RT-qPCR. Annotation columns are as follows: *FC*, peak log_2_ fold change; *GS*, gene symbol; *FGN*, full gene name. **Figure S2.** Cytokines differentially expressed greater than 2-fold (log_2_ fold change ≥ 1) between DBA/2 and C57BL/6 mice at day 15 following infection with *C. immitis*. The Mouse Common Cytokines Gene Array from SABiosciences was used to detect cytokine expression. All cytokines depicted were expressed to a greater extent in DBA/2 compared to C57BL/6 mice. Gene symbol abbreviations are defined as follows: *IFNG*, interferon gamma; *KITL*, KIT ligand; *AIF1*, allograft inflammatory factor 1; *IL-17*, interleukin-17A. **Figure S3.** Confirmation of gene expression differences by RT-qPCR between DBA/2 and C57BL/6 mice at day 10 (**A**) and day 16 (**B**) following *C. immitis* infection. The fold change for each gene, calculated by dividing the expression level in DBA/2 mice by the expression level in C57BL/6 mice is presented for RT-qPCR data (grey bars) for comparison to microarray data (black bars). At day 10 gene expression was assessed in three independent samples from each mice strain and at day 16 using 1 sample from C57BL/6 mice and 3 samples from DBA/2 mice. RT-qPCR gene expression data (2^-∆∆CT^) was averaged within mouse strains at each time point and used to calculate log_2_ fold change values between strains for direct comparison to microarray data. A log_2_ fold change of 1 equates to an actual fold change of 2. A positive fold change indicates the gene was expressed to a greater extent in DBA/2 mice. An asterisk (*) indicates that the gene was significantly differentially expressed (*p* <0.05, *t*-test) between mice strains at day 10. Statistics could not be generated at day 16 since there was only one sample in the C57BL/6 group. Click here for file

Additional file 2 **Table S1.** Genes significantly differentially expressed with a fold change ≥ 2 or ≤ -2 between DBA/2 and C57BL/6 mice at any time point following infection with *C. immitis* (N=1334) were significantly over-represented in four KEGG pathways. **Table S2.** Genes significantly differentially expressed with a fold change ≥ 2 or ≤ -2 between DBA/2 and C57BL/6 mice at any time point following infection with *C. immitis* (N=1334) were significantly over-represented in a large number of gene ontology terms. Click here for file

## References

[B1] Fisher MC Koenig GL White TJ Taylor JW Molecular and phenotypic description of Coccidioides posadasii sp. nov., previously recognized as the non-California population of Coccidioides immitis Mycologia 2002 94 73 84 10.2307/3761847 21156479

[B2] Laniado-Laborin R Expanding understanding of epidemiology of coccidioidomycosis in the Western hemisphere Ann N Y Acad Sci 2007 1111 19 34 10.1196/annals.1406.004 17395731

[B3] Kirkland TN Fierer J Coccidioidomycosis: a reemerging infectious disease Emerg Infect Dis 1996 2 192 199 10.3201/eid0203.960305 8903229 PMC2626789

[B4] Valdivia L Nix D Wright M Lindberg E Fagan T Lieberman D Stoffer T Ampel NM Galgiani JN Coccidioidomycosis as a common cause of community-acquired pneumonia Emerg Infect Dis 2006 12 958 962 10.3201/eid1206.060028 16707052 PMC3373055

[B5] Ampel NM Dols CL Galgiani JN Coccidioidomycosis during human immunodeficiency virus infection: results of a prospective study in a coccidioidal endemic area Am J Med 1993 94 235 240 10.1016/0002-9343(93)90054-S 8095771

[B6] Bergstrom L Yocum DE Ampel NM Villanueva I Lisse J Gluck O Tesser J Posever J Miller M Araujo J Increased risk of coccidioidomycosis in patients treated with tumor necrosis factor alpha antagonists Arthritis Rheum 2004 50 1959 1966 10.1002/art.20454 15188373

[B7] Pappagianis D Epidemiology of coccidioidomycosis Curr Top Med Mycol 1988 2 199 238 10.1007/978-1-4612-3730-3_6 3288356

[B8] Gray GC Fogle EF Albright KL Risk factors for primary pulmonary coccidioidomycosis hospitalizations among United States Navy and Marine Corps personnel, 1981–1994 Am J Trop Med Hyg 1998 58 309 312 9546408 10.4269/ajtmh.1998.58.309

[B9] Smith CE Saito MT Simons SA Pattern of 39,500 serologic tests in coccidioidomycosis J Am Med Assoc 1956 160 546 552 10.1001/jama.1956.02960420026008 13286095

[B10] Kirkland TN Fierer J Inbred mouse strains differ in resistance to lethal Coccidioides immitis infection Infect Immun 1983 40 912 916 6852925 10.1128/iai.40.3.912-916.1983 PMC348138

[B11] Fierer J Walls L Wright F Kirkland TN Genes influencing resistance to Coccidioides immitis and the interleukin-10 response map to chromosomes 4 and 6 in mice Infect Immun 1999 67 2916 2919 10338499 10.1128/iai.67.6.2916-2919.1999 PMC96600

[B12] Fierer J Walls L Eckmann L Yamamoto T Kirkland TN Importance of interleukin-10 in genetic susceptibility of mice to Coccidioides immitis Infect Immun 1998 66 4397 4402 9712793 10.1128/iai.66.9.4397-4402.1998 PMC108531

[B13] Moore KW de Waal Malefyt R Coffman RL O'Garra A Interleukin-10 and the interleukin-10 receptor Annu Rev Immunol 2001 19 683 765 10.1146/annurev.immunol.19.1.683 11244051

[B14] Waddell SJ Popper SJ Rubins KH Griffiths MJ Brown PO Levin M Relman DA Dissecting interferon-induced transcriptional programs in human peripheral blood cells PLoS One 2010 5 e9753 10.1371/journal.pone.0009753 20339534 PMC2842296

[B15] Dennis G Jr Sherman BT Hosack DA Yang J Gao W Lane HC Lempicki RA DAVID: Database for Annotation, Visualization, and Integrated Discovery Genome Biol 2003 4 P3 10.1186/gb-2003-4-5-p3 12734009 PMC193660

[B16] Maere S Heymans K Kuiper M BiNGO: a Cytoscape plugin to assess overrepresentation of gene ontology categories in biological networks Bioinformatics 2005 21 3448 3449 10.1093/bioinformatics/bti551 15972284

[B17] Thomas MJ Seto E Unlocking the mechanisms of transcription factor YY1: are chromatin modifying enzymes the key? Gene 1999 236 197 208 10.1016/S0378-1119(99)00261-9 10452940

[B18] Ratcliffe PJ From erythropoietin to oxygen: hypoxia-inducible factor hydroxylases and the hypoxia signal pathway Blood Purification 2002 20 445 450 10.1159/000065201 12207089

[B19] Semenza GL Hypoxia-inducible factor 1: master regulator of O2 homeostasis Curr Opin Genet Dev 1998 8 588 594 10.1016/S0959-437X(98)80016-6 9794818

[B20] Viemann D Schmidt M Tenbrock K Schmid S Müller V Klimmek K Ludwig S Roth J Goebeler M The contact allergen nickel triggers a unique inflammatory and proangiogenic gene expression pattern via activation of NF-kappaB and hypoxia-inducible factor-1alpha Journal of Immunology (Baltimore, Md.: 1950) 2007 178 3198 3207 10.4049/jimmunol.178.5.3198 17312168

[B21] Costa-Pereira AP Tininini S Strobl B Alonzi T Schlaak JF Is'harc H Gesualdo I Newman SJ Kerr IM Poli V Mutational switch of an IL-6 response to an interferon-gamma-like response Proceedings of the National Academy of Sciences of the United States of America 2002 99 8043 8047 10.1073/pnas.122236099 12060750 PMC123017

[B22] Lieberman LA Banica M Reiner SL Hunter CA STAT1 plays a critical role in the regulation of antimicrobial effector mechanisms, but not in the development of Th1-type responses during toxoplasmosis Journal of Immunology (Baltimore, Md.: 1950) 2004 172 457 463 10.4049/jimmunol.172.1.457 14688355

[B23] Robertson G Hirst M Bainbridge M Bilenky M Zhao Y Zeng T Euskirchen G Bernier B Varhol R Delaney A Genome-wide profiles of STAT1 DNA association using chromatin immunoprecipitation and massively parallel sequencing Nature Methods 2007 4 651 657 10.1038/nmeth1068 17558387

[B24] Oliva J Bardag-Gorce F Lin A French BA French SW The role of cytokines in UbD promoter regulation and Mallory-Denk body-like aggresomes Experimental and Molecular Pathology 2010 89 1 8 10.1016/j.yexmp.2010.04.001 20433827 PMC2900536

[B25] Lukasiak S Schiller C Oehlschlaeger P Schmidtke G Krause P Legler DF Autschbach F Schirmacher P Breuhahn K Groettrup M Proinflammatory cytokines cause FAT10 upregulation in cancers of liver and colon Oncogene 2008 27 6068 6074 10.1038/onc.2008.201 18574467

[B26] Farber JM HuMig: a new human member of the chemokine family of cytokines Biochem Biophys Res Commun 1993 192 223 230 10.1006/bbrc.1993.1403 8476424

[B27] Padovan E Spagnoli GC Ferrantini M Heberer M IFN-alpha2a induces IP-10/CXCL10 and MIG/CXCL9 production in monocyte-derived dendritic cells and enhances their capacity to attract and stimulate CD8+ effector T cells J Leukoc Biol 2002 71 669 676 11927654

[B28] Nickoloff BJ Riser BL Mitra RS Dixit VM Varani J Inhibitory effect of gamma interferon on cultured human keratinocyte thrombospondin production, distribution, and biologic activities J Invest Dermatol 1988 91 213 218 10.1111/1523-1747.ep12465005 2457631

[B29] Magee DM Cox RA Roles of gamma interferon and interleukin-4 in genetically determined resistance to Coccidioides immitis Infect Immun 1995 63 3514 3519 7642285 10.1128/iai.63.9.3514-3519.1995 PMC173486

[B30] Vinh DC Masannat F Dzioba RB Galgiani JN Holland SM Refractory disseminated coccidioidomycosis and mycobacteriosis in interferon-gamma receptor 1 deficiency Clin Infect Dis 2009 49 e62 e65 10.1086/605532 19681704 PMC2730428

[B31] Vinh DC Schwartz B Hsu AP Miranda DJ Valdez PA Fink D Lau KP Long-Priel D Kuhns DB Uzel G Interleukin-12 receptor beta1 deficiency predisposing to disseminated Coccidioidomycosis Clin Infect Dis 2011 52 e99 e102 10.1093/cid/ciq215 21258095 PMC3060907

[B32] Stark GR Kerr IM Williams BR Silverman RH Schreiber RD How cells respond to interferons Annu Rev Biochem 1998 67 227 264 10.1146/annurev.biochem.67.1.227 9759489

[B33] Kalveram B Schmidtke G Groettrup M The ubiquitin-like modifier FAT10 interacts with HDAC6 and localizes to aggresomes under proteasome inhibition J Cell Sci 2008 121 4079 4088 10.1242/jcs.035006 19033385

[B34] Gong P Canaan A Wang B Leventhal J Snyder A Nair V Cohen CD Kretzler M D'Agati V Weissman S The ubiquitin-like protein FAT10 mediates NF-kappaB activation J Am Soc Nephrol 2010 21 316 326 10.1681/ASN.2009050479 19959714 PMC2834541

[B35] Raasi S Schmidtke G Groettrup M The ubiquitin-like protein FAT10 forms covalent conjugates and induces apoptosis J Biol Chem 2001 276 35334 35343 10.1074/jbc.M105139200 11445583

[B36] Xanthou G Duchesnes CE Williams TJ Pease JE CCR3 functional responses are regulated by both CXCR3 and its ligands CXCL9, CXCL10 and CXCL11 Eur J Immunol 2003 33 2241 2250 10.1002/eji.200323787 12884299

[B37] Singal DP Ye M Quadr SA Major histocompatibility-encoded human proteasome LMP2. Genomic organization and a new form of mRNA J Biol Chem 1995 270 1966 1970 10.1074/jbc.270.4.1966 7829535

[B38] Mishto M Bonafe M Salvioli S Olivieri F Franceschi C Age dependent impact of LMP polymorphisms on TNFalpha-induced apoptosis in human peripheral blood mononuclear cells Exp Gerontol 2002 37 301 308 10.1016/S0531-5565(01)00196-6 11772516

[B39] Zimmerer JM Lesinski GB Radmacher MD Ruppert A Carson WE 3rd STAT1-dependent and STAT1-independent gene expression in murine immune cells following stimulation with interferon-alpha Cancer Immunol Immunother 2007 56 1845 1852 10.1007/s00262-007-0329-9 17503042 PMC11030667

[B40] Schmidtke G Eggers M Ruppert T Groettrup M Koszinowski UH Kloetzel PM Inactivation of a defined active site in the mouse 20S proteasome complex enhances major histocompatibility complex class I antigen presentation of a murine cytomegalovirus protein J Exp Med 1998 187 1641 1646 10.1084/jem.187.10.1641 9584142 PMC2212286

[B41] Taylor GA IRG proteins: key mediators of interferon-regulated host resistance to intracellular pathogens Cell Microbiol 2007 9 1099 1107 10.1111/j.1462-5822.2007.00916.x 17359233

[B42] MacMicking JD Taylor GA McKinney JD Immune control of tuberculosis by IFN-gamma-inducible LRG-47 Science 2003 302 654 659 10.1126/science.1088063 14576437

[B43] Butcher BA Greene RI Henry SC Annecharico KL Weinberg JB Denkers EY Sher A Taylor GA p47 GTPases regulate Toxoplasma gondii survival in activated macrophages Infect Immun 2005 73 3278 3286 10.1128/IAI.73.6.3278-3286.2005 15908352 PMC1111824

[B44] Henry SC Traver M Daniell X Indaram M Oliver T Taylor GA Regulation of macrophage motility by Irgm1 Journal of Leukocyte Biology 2010 87 333 343 10.1189/jlb.0509299 19920210 PMC2812558

[B45] Singh SB Davis AS Taylor GA Deretic V Human IRGM induces autophagy to eliminate intracellular mycobacteria Science 2006 313 1438 1441 10.1126/science.1129577 16888103

[B46] Okamoto T Gohil K Finkelstein EI Bove P Akaike T van der Vliet A Multiple contributing roles for NOS2 in LPS-induced acute airway inflammation in mice Am J Physiol Lung Cell Mol Physiol 2004 286 L198 L209 12972406 10.1152/ajplung.00136.2003

[B47] Wang Y Barbacioru C Hyland F Xiao W Hunkapiller KL Blake J Chan F Gonzalez C Zhang L Samaha RR Large scale real-time PCR validation on gene expression measurements from two commercial long-oligonucleotide microarrays BMC Genomics 2006 7 59 10.1186/1471-2164-7-59 16551369 PMC1435885

[B48] Lawler J Sunday M Thibert V Duquette M George EL Rayburn H Hynes RO Thrombospondin-1 is required for normal murine pulmonary homeostasis and its absence causes pneumonia J Clin Invest 1998 101 982 992 10.1172/JCI1684 9486968 PMC508649

[B49] Shubitz LF Dial SM Perrill R Casement R Galgiani JN Vaccine-induced cellular immune responses differ from innate responses in susceptible and resistant strains of mice infected with Coccidioides posadasii Infect Immun 2008 76 5553 5564 10.1128/IAI.00885-08 18852250 PMC2583549

[B50] Johnson LA Prevo R Clasper S Jackson DG Inflammation-induced uptake and degradation of the lymphatic endothelial hyaluronan receptor LYVE-1 J Biol Chem 2007 282 33671 33680 10.1074/jbc.M702889200 17884820

[B51] Gale NW Prevo R Espinosa J Ferguson DJ Dominguez MG Yancopoulos GD Thurston G Jackson DG Normal lymphatic development and function in mice deficient for the lymphatic hyaluronan receptor LYVE-1 Mol Cell Biol 2007 27 595 604 10.1128/MCB.01503-06 17101772 PMC1800809

[B52] Fandrey J Gorr TA Gassmann M Regulating cellular oxygen sensing by hydroxylation Cardiovasc Res 2006 71 642 651 10.1016/j.cardiores.2006.05.005 16780822

[B53] van Uden P Kenneth NS Rocha S Regulation of hypoxia-inducible factor-1alpha by NF-kappaB Biochem J 2008 412 477 484 10.1042/BJ20080476 18393939 PMC2474706

[B54] Lowenthal JW Ballard DW Bogerd H Bohnlein E Greene WC Tumor necrosis factor-alpha activation of the IL-2 receptor-alpha gene involves the induction of kappa B-specific DNA binding proteins J Immunol 1989 142 3121 3128 2785134

[B55] Galgiani JN Ampel NM Blair JE Catanzaro A Johnson RH Stevens DA Williams PL Coccidioidomycosis Clin Infect Dis 2005 41 1217 1223 10.1086/496991 16206093

[B56] Miller MB Hendren R Gilligan PH Posttransplantation disseminated coccidioidomycosis acquired from donor lungs J Clin Microbiol 2004 42 2347 2349 10.1128/JCM.42.5.2347-2349.2004 15131231 PMC404664

[B57] Nizet V Johnson RS Interdependence of hypoxic and innate immune responses Nat Rev Immunol 2009 9 609 617 10.1038/nri2607 19704417 PMC4343208

[B58] Cox RA Magee DM Production of tumor necrosis factor alpha, interleukin-1 alpha, and interleukin-6 during murine coccidioidomycosis Infect Immun 1995 63 4178 4180 7558338 10.1128/iai.63.10.4178-4180.1995 PMC173589

[B59] Fierer J Waters C Walls L Both CD4+ and CD8+ T cells can mediate vaccine-induced protection against Coccidioides immitis infection in mice J Infect Dis 2006 193 1323 1331 10.1086/502972 16586371

[B60] Jacobs MD Harrison SC Structure of an IkappaBalpha/NF-kappaB complex Cell 1998 95 749 758 10.1016/S0092-8674(00)81698-0 9865693

[B61] Ji Y Zhang W Th17 cells: positive or negative role in tumor? Cancer Immunol Immunother 2010 59 979 987 10.1007/s00262-010-0849-6 20352428 PMC11031007

[B62] Hung CY Gonzalez A Wuthrich M Klein BS Cole GT Vaccine immunity to coccidioidomycosis occurs by early activation of three signal pathways of T helper cell response (Th1, Th2, and Th17) Infect Immun 2011 79 4511 4522 10.1128/IAI.05726-11 21859851 PMC3257932

[B63] Kuberski TT Servi RJ Rubin PJ Successful treatment of a critically ill patient with disseminated coccidioidomycosis, using adjunctive interferon-gamma Clin Infect Dis 2004 38 910 912 10.1086/382075 14999639

[B64] Oshlack A Robinson MD Young MD From RNA-seq reads to differential expression results Genome Biol 2010 11 220 10.1186/gb-2010-11-12-220 21176179 PMC3046478

[B65] Jimenez Mdel P Walls L Fierer J High levels of interleukin-10 impair resistance to pulmonary coccidioidomycosis in mice in part through control of nitric oxide synthase 2 expression Infect Immun 2006 74 3387 3395 10.1128/IAI.01985-05 16714569 PMC1479230

[B66] Bolstad BM Collin F Brettschneider J Simpson K Cope L Irizarry R Speed TP Gentleman R, Carey V, Huber W, Irizarry R, Dutoit S Quality Assessment of Affymetrix GeneChip Data Bioinformatics and Computational Biology Solutions Using R and Bioconductor 2005 Heidelberg: Springer 33 47

[B67] Wu Z Irizarry RA Gentleman R Martinez-Murillo F Spencer F A Model-Based Background Adjustment for Oligonucleotide Expression Arrays Journal of the American Statistical Association 2004 99 909 917 10.1198/016214504000000683

[B68] Hubbell E Liu W-M Mei R Robust estimators for expression analysis Bioinformatics (Oxford, England) 2002 18 1585 1592 10.1093/bioinformatics/18.12.1585 12490442

[B69] Hastings JM Jackson KS Mavrogianis PA Fazleabas AT The Estrogen Early Response Gene FOS Is Altered in a Baboon Model of Endometriosis Biology of Reproduction 2006 75 176 182 10.1095/biolreprod.106.052852 16672717

[B70] Kanehisa M Goto S Furumichi M Tanabe M Hirakawa M KEGG for representation and analysis of molecular networks involving diseases and drugs Nucleic Acids Research 2010 38 D355 D360 10.1093/nar/gkp896 19880382 PMC2808910

[B71] Benjamini Y Hochberg Y Controlling the False Discovery Rate: A Practical and Powerful Approach to Multiple Testing Journal of the Royal Statistical Society. Series B (Methodological) 1995 57 289 300

[B72] Shannon P Markiel A Ozier O Baliga NS Wang JT Ramage D Amin N Schwikowski B Ideker T Cytoscape: a software environment for integrated models of biomolecular interaction networks Genome Res 2003 13 2498 2504 10.1101/gr.1239303 14597658 PMC403769

[B73] Yin R Tian F Frankenberger B de Angelis MH Stoeger T Selection and evaluation of stable housekeeping genes for gene expression normalization in carbon nanoparticle-induced acute pulmonary inflammation in mice Biochemical and Biophysical Research Communications 2010 399 531 536 10.1016/j.bbrc.2010.07.104 20678479

[B74] Konstantinidou V Covas MI Munoz-Aguayo D Khymenets O de la Torre R Saez G Tormos Mdel C Toledo E Marti A Ruiz-Gutierrez V In vivo nutrigenomic effects of virgin olive oil polyphenols within the frame of the Mediterranean diet: a randomized controlled trial FASEB J 2010 24 2546 2557 10.1096/fj.09-148452 20179144

[B75] Rieu I Powers SJ Real-time quantitative RT-PCR: design, calculations, and statistics Plant Cell 2009 21 1031 1033 10.1105/tpc.109.066001 19395682 PMC2685626

