# Supplementary material for: Factors regulated by interferon gamma and hypoxia-inducible factor 1A contribute to responses that protect mice from Coccidioides immitis infection
Source: BMC Microbiol. 2012 Sep 24;12:218. doi: 10.1186/1471-2180-12-218 (PMC3528620; doi:10.1186/1471-2180-12-218)
Supplement: Additional file 1 — Figure S1. A heatmap depicting log2 fold changes between pre- (Day 0) and post- (Days 10, 14 and 16) infection time points for the top 100 modulated genes depicted in Figure 2. The log2 fold change scale is indicated at the bottom of the heatmap, where red shading indicates upregulation post- versus pre-infection and blue shading represents downregulation. Hierarchical clustering of genes based on their expression profiles over the time course was performed by calculating distances using the Pearson correlation metric and then clustering these distances using the average linkage method. The expression of genes marked with an asterisk (*) was confirmed by RT-qPCR. Annotation columns are as follows: FC, peak log2 fold change; GS, gene symbol; FGN, full gene name. Figure S2. Cytokines differentially expressed greater than 2-fold (log2 fold change ≥ 1) between DBA/2 and C57BL/6 mice at day 15 following infection with C. immitis. The Mouse Common Cytokines Gene Array from SABiosciences was used to detect cytokine expression. All cytokines depicted were expressed to a greater extent in DBA/2 compared to C57BL/6 mice. Gene symbol abbreviations are defined as follows: IFNG, interferon gamma; KITL, KIT ligand; AIF1, allograft inflammatory factor 1; IL-17, interleukin-17A. Figure S3. Confirmation of gene expression differences by RT-qPCR between DBA/2 and C57BL/6 mice at day 10 (A) and day 16 (B) following C. immitis infection. The fold change for each gene, calculated by dividing the expression level in DBA/2 mice by the expression level in C57BL/6 mice is presented for RT-qPCR data (grey bars) for comparison to microarray data (black bars). At day 10 gene expression was assessed in three independent samples from each mice strain and at day 16 using 1 sample from C57BL/6 mice and 3 samples from DBA/2 mice. RT-qPCR gene expression data (2-∆∆CT) was averaged within mouse strains at each time point and used to calculate log2 fold change values between strains for direct compari [file 1471-2180-12-218-S1.doc]

**SUPPLEMENTAL FIGURES**

**Supplemental Figure 1.** A heatmap depicting log2 fold changes between pre- (Day 0) and post- (Days 10, 14 and 16) infection time points for the top 100 modulated genes depicted in **Figure 2**. The log2 fold change scale is indicated at the bottom of the heatmap, where red shading indicates upregulation post- versus pre-infection and blue shading represents downregulation. Hierarchical clustering of genes based on their expression profiles over the time course was performed by calculating distances using the Pearson correlation metric and then clustering these distances using the average linkage method. The expression of genes marked with an asterisk (*) was confirmed by RT-qPCR. Annotation columns are as follows: *FC*, peak log2 fold change; *GS*, gene symbol; *FGN*, full gene name.


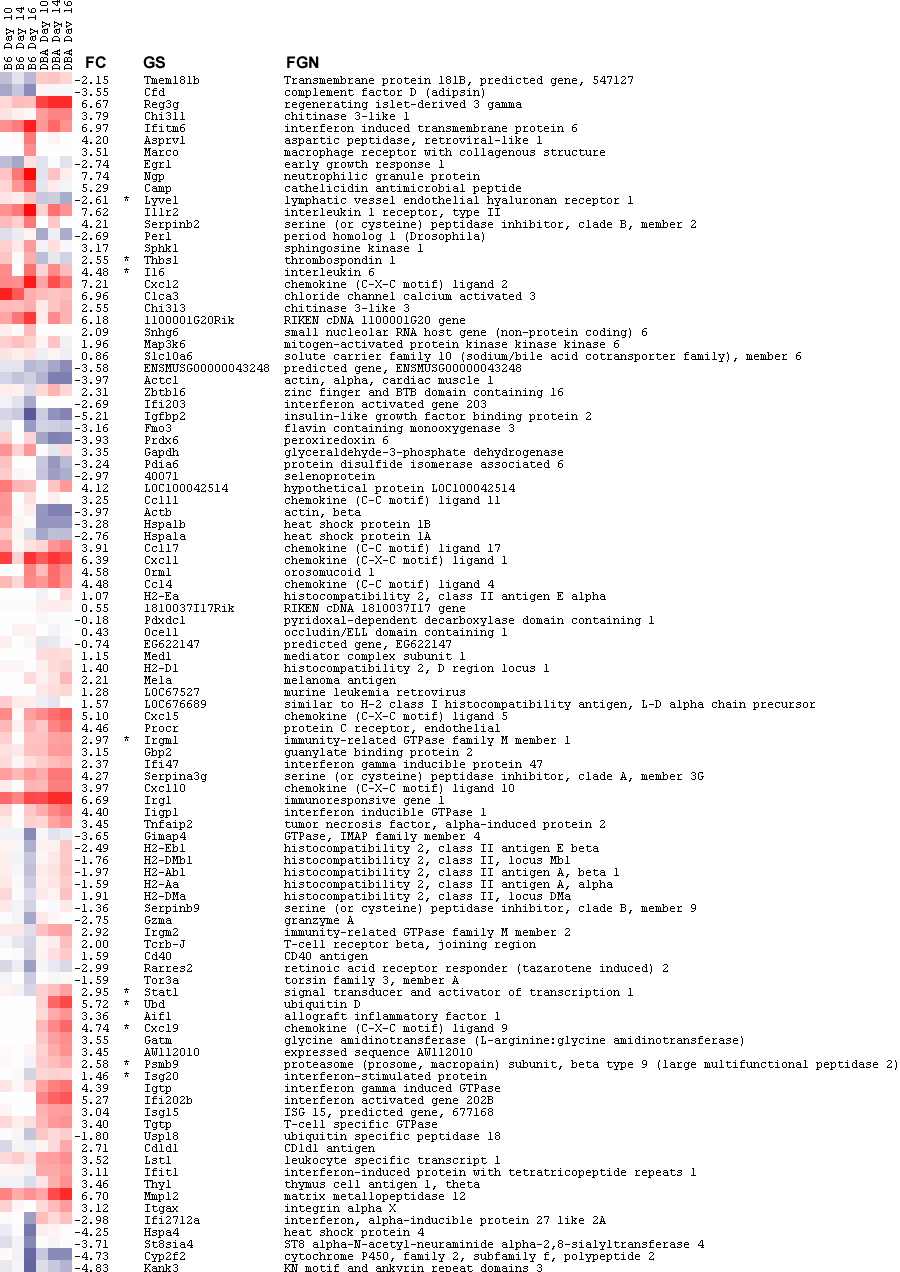


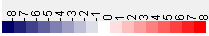


**Supplemental Figure 2.** Cytokines differentially expressed greater than 2-fold (log2 fold change ≥ 1) between DBA/2 and C57BL/6 mice at day 15 following infection with *C. immitis*. The Mouse Common Cytokines Gene Array from SABiosciences was used to detect cytokine expression. All cytokines depicted were expressed to a greater extent in DBA/2 compared to C57BL/6 mice. Gene symbol abbreviations are defined as follows: *IFNG*, interferon gamma; *KITL*, KIT ligand; *AIF1*, allograft inflammatory factor 1; *IL-17*, interleukin-17A.

**
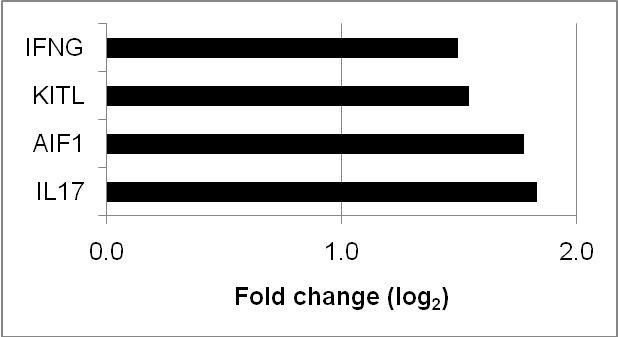
**

**Supplemental Figure 3. Confirmation of gene expression differences by RT-qPCR between DBA/2 and C57BL/6 mice at day 10 (A) and day 16 (B) following *C. immitis* infection. The fold change for each gene, calculated by dividing the expression level in DBA/2 mice by the expression level in C57BL/6 mice is presented for RT-qPCR data (grey bars) for comparison to microarray data (black bars). At day 10 gene expression was assessed in three independent samples from each mice strain and at day 16 using 1 sample from C57BL/6 mice and 3 samples from DBA/2 mice. RT-qPCR gene expression data (2-∆∆CT­) was averaged within mouse strains at each time point and used to calculate log2 fold change values between strains for direct comparison to microarray data. A log2 fold change of 1 equates to an actual fold change of 2. A positive fold change indicates the gene was expressed to a greater extent in DBA/2 mice. An asterisk (*) indicates that the gene was significantly differentially expressed (*p* <0.05, *t*-test) between mice strains at day 10. Statistics could not be generated at day 16 since there was only one sample in the C57BL/6 group.**

| **A)** | **B)** |
| --- | --- |
| 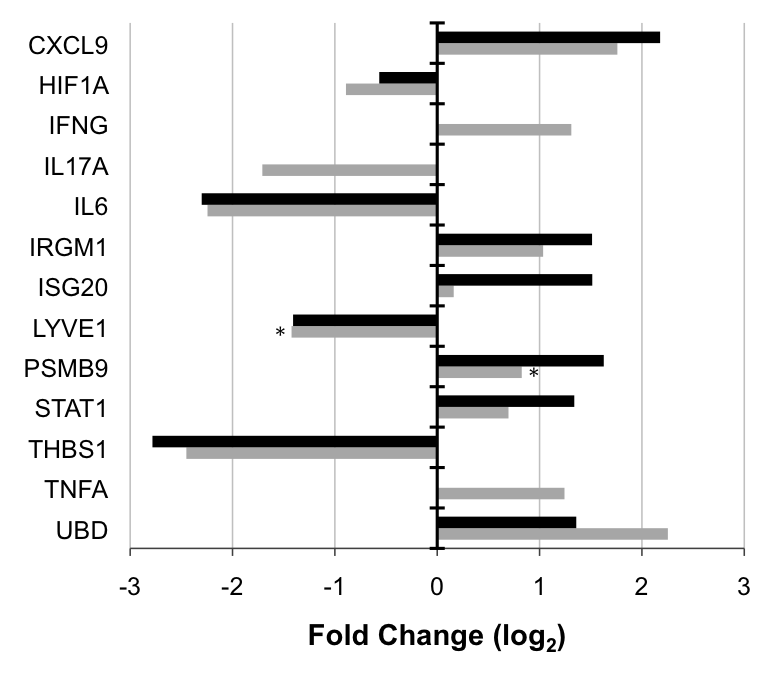 | 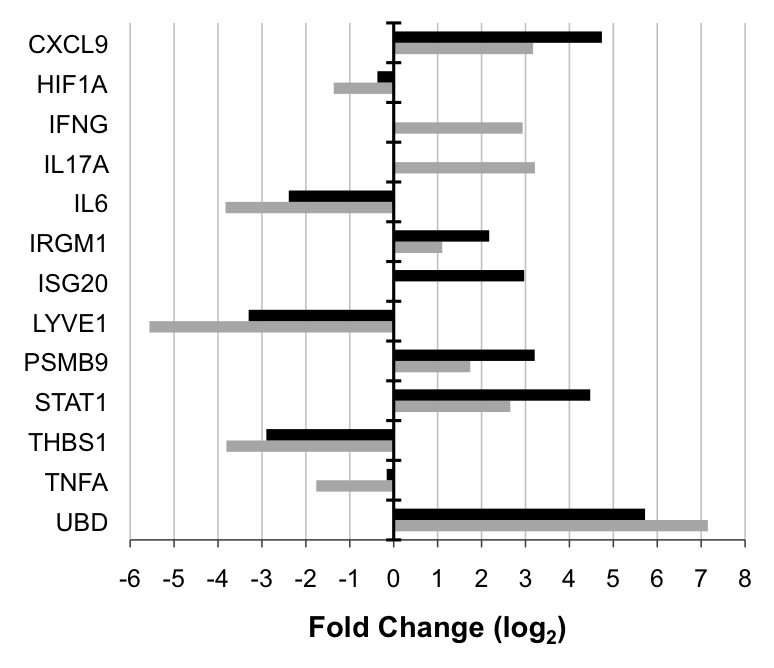 |
