# Supplementary material for: Factors regulated by interferon gamma and hypoxia-inducible factor 1A contribute to responses that protect mice from Coccidioides immitis infection
Source: BMC Microbiol. 2012 Sep 24;12:218. doi: 10.1186/1471-2180-12-218 (PMC3528620; doi:10.1186/1471-2180-12-218)
Supplement: Additional file 2 — Table S1. Genes significantly differentially expressed with a fold change ≥ 2 or ≤ -2 between DBA/2 and C57BL/6 mice at any time point following infection with C. immitis (N=1334) were significantly over-represented in four KEGG pathways. Table S2. Genes significantly differentially expressed with a fold change ≥ 2 or ≤ -2 between DBA/2 and C57BL/6 mice at any time point following infection with C. immitis (N=1334) were significantly over-represented in a large number of gene ontology terms. [file 1471-2180-12-218-S2.doc]

**SUPPLEMENTARY TABLES**

| **Supplementary Table 1.** Genes significantly differentially expressed with a fold change ≥ 2 or ≤ -2 between DBA/2 and C57BL/6 mice at any time point following infection with *C. immitis* (N=1334) were significantly over-represented in four KEGG pathways.^1^ | | | |
| --- | --- | --- | --- |
| **Pathway** | **Count** | **KEGG ID** | **FDR *p*-value** |
| Hematopoietic cell lineage | 28 | mmu04640 | 1.9E-2 |
| Viral myocarditis | 26 | mmu05416 | 2.1E-2 |
| Cytokine-cytokine receptor interaction | 51 | mmu04060 | 2.6E-2 |
| Chemokine signaling pathway | 42 | mmu04062 | 3.2E-2 |
| ^1^Column headings are as follows: *Count*, refers to the number of differentially expressed genes associated with each pathway; *KEGG ID*, refers to the pathway identification number associated with the Kyoto Encyclopedia of Genes and Genomes, *FDR p-value*, is the false discovery rate corrected *p*-value using the BH method and only pathways with an FDR *p*-value <0.05 are presented. | | | |

| **Supplemental Table 2.** Genes significantly differentially expressed with a fold change ≥ 2 or ≤ -2 between DBA/2 and C57BL/6 mice at any time point following infection with *C. immitis* (N=1334) were significantly over-represented in a large number of gene ontology terms.^1^ | | | |
| --- | --- | --- | --- |
| **GO ID** | **GO Term** | **Count** | **FDR *p*-value** |
| 6955 | immune response | 77 | 6.51E-09 |
| 2376 | immune system process | 128 | 2.83E-08 |
| 6952 | defense response | 77 | 2.45E-07 |
| 50896 | response to stimulus | 296 | 2.45E-06 |
| 6954 | inflammatory response | 49 | 2.88E-05 |
| 6950 | response to stress | 171 | 5.27E-05 |
| 42221 | response to chemical stimulus | 158 | 5.27E-05 |
| 19882 | antigen processing and presentation | 21 | 6.38E-05 |
| 19884 | antigen processing and presentation of exogenous antigen | 15 | 2.26E-04 |
| 9611 | response to wounding | 66 | 6.63E-04 |
| 48002 | antigen processing and presentation of peptide antigen | 14 | 1.26E-03 |
| 10033 | response to organic substance | 99 | 1.26E-03 |
| 9607 | response to biotic stimulus | 51 | 1.28E-03 |
| 2478 | antigen processing and presentation of exogenous peptide antigen | 12 | 2.24E-03 |
| 2682 | regulation of immune system process | 66 | 2.24E-03 |
| 45059 | positive thymic T cell selection | 7 | 2.66E-03 |
| 1817 | regulation of cytokine production | 32 | 4.64E-03 |
| 2711 | positive regulation of T cell mediated immunity | 8 | 5.42E-03 |
| 10466 | negative regulation of peptidase activity | 15 | 7.98E-03 |
| 43368 | positive T cell selection | 7 | 8.35E-03 |
| 51707 | response to other organism | 39 | 9.73E-03 |
| 1914 | regulation of T cell mediated cytotoxicity | 8 | 1.11E-02 |
| 2709 | regulation of T cell mediated immunity | 9 | 1.11E-02 |
| 52547 | regulation of peptidase activity | 23 | 1.11E-02 |
| 51239 | regulation of multicellular organismal process | 154 | 1.11E-02 |
| 45087 | innate immune response | 22 | 1.11E-02 |
| 1819 | positive regulation of cytokine production | 18 | 1.33E-02 |
| 6935 | chemotaxis | 26 | 1.37E-02 |
| 42330 | taxis | 26 | 1.37E-02 |
| 1916 | positive regulation of T cell mediated cytotoxicity | 7 | 1.62E-02 |
| 45061 | thymic T cell selection | 8 | 1.86E-02 |
| 2684 | positive regulation of immune system process | 44 | 1.90E-02 |
| 50776 | regulation of immune response | 39 | 1.94E-02 |
| 2495 | antigen processing and presentation of peptide antigen via MHC class II | 8 | 2.67E-02 |
| 2824 | positive regulation of adaptive immune response based on somatic recombination of immune receptors built from immunoglobulin superfamily domains | 11 | 2.67E-02 |
| 2821 | positive regulation of adaptive immune response | 11 | 2.67E-02 |
| 2822 | regulation of adaptive immune response based on somatic recombination of immune receptors built from immunoglobulin superfamily domains | 16 | 2.67E-02 |
| 8210 | estrogen metabolic process | 7 | 2.67E-02 |
| 51346 | negative regulation of hydrolase activity | 16 | 2.67E-02 |
| 2819 | regulation of adaptive immune response | 16 | 2.67E-02 |
| 2504 | antigen processing and presentation of peptide or polysaccharide antigen via MHC class II | 9 | 2.67E-02 |
| 19886 | antigen processing and presentation of exogenous peptide antigen via MHC class II | 8 | 2.67E-02 |
| 51704 | multi-organism process | 52 | 2.67E-02 |
| 51094 | positive regulation of developmental process | 62 | 2.67E-02 |
| 50793 | regulation of developmental process | 116 | 3.14E-02 |
| 1959 | regulation of cytokine-mediated signaling pathway | 5 | 3.14E-02 |
| 50778 | positive regulation of immune response | 27 | 3.14E-02 |
| 9056 | catabolic process | 108 | 3.28E-02 |
| 34097 | response to cytokine stimulus | 15 | 3.79E-02 |
| 10743 | regulation of macrophage derived foam cell differentiation | 4 | 3.79E-02 |
| 2699 | positive regulation of immune effector process | 14 | 3.79E-02 |
| 60326 | cell chemotaxis | 11 | 4.95E-02 |
| 50900 | leukocyte migration | 14 | 5.00E-02 |
| ^1^*GO ID*, refers to gene ontology identification number; *Count*, refers to the number of differentially expressed genes associated with each GO term; *FDR p-value*, is the false discovery rate corrected *p*-value using the BH method and GO terms with an FDR *p*-value <0.05 are presented. | | | |
